# LRP5 Regulates HIF-1α Stability via Interaction with PHD2 in Ischemic Myocardium

**DOI:** 10.3390/ijms22126581

**Published:** 2021-06-19

**Authors:** Sujin Ju, Leejin Lim, Kwanhwan Wi, Changwon Park, Young-Jae Ki, Dong-Hyun Choi, Heesang Song

**Affiliations:** 1 Department of Biochemistry and Molecular Biology, Chosun University School of Medicine, Gwangju 61452, Korea; sujin1720@chosun.ac.kr (S.J.); gfvlmnz12@naver.com (K.W.); 2 Cancer Mutation Research Center, Chosun University, Gwangju 61452, Korea; leejinlim@gmail.com; 3 Department of Molecular & Cellular Physiology, Louisiana State University Health Sciences Center, Shreveport, LA 71103, USA; cpar13@lhuhsc.edu; 4 Department of Internal Medicine, Chosun University School of Medicine, Gwangju 61452, Korea; bigpapi0427@chosun.ac.kr (Y.-J.K.); dhchoi@chosun.ac.kr (D.-H.C.)

**Keywords:** low-density lipoprotein receptor-related protein 5, myocardial infarction, hypoxia-inducible factor-1α, HIF-prolyl hydroxylases 2

## Abstract

Low-density lipoprotein receptor-related protein 5 (LRP5) has been studied as a co-receptor for Wnt/β-catenin signaling. However, its role in the ischemic myocardium is largely unknown. Here, we show that LRP5 may act as a negative regulator of ischemic heart injury via its interaction with prolyl hydroxylase 2 (PHD2), resulting in hypoxia-inducible factor-1α (HIF-1α) degradation. Overexpression of LRP5 in cardiomyocytes promoted hypoxia-induced apoptotic cell death, whereas LRP5-silenced cardiomyocytes were protected from hypoxic insult. Gene expression analysis (mRNA-seq) demonstrated that overexpression of LRP5 limited the expression of HIF-1α target genes. LRP5 promoted HIF-1α degradation, as evidenced by the increased hydroxylation and shorter stability of HIF-1α under hypoxic conditions through the interaction between LRP5 and PHD2. Moreover, the specific phosphorylation of LRP5 at T1492 and S1503 is responsible for enhancing the hydroxylation activity of PHD2, resulting in HIF-1α degradation, which is independent of Wnt/β-catenin signaling. Importantly, direct myocardial delivery of adenoviral constructs, silencing LRP5 in vivo, significantly improved cardiac function in infarcted rat hearts, suggesting the potential value of LRP5 as a new target for ischemic injury treatment.

## 1. Introduction

Hypoxia, the state of oxygen deficiency in tissues or organs, is a common feature of cardiovascular diseases such as myocardial infarction (MI) [1,2]. MI results from blood flow disruption, leading to irreversible myocardial tissue damage and permanent functional deficits [3]. Hypoxia-induced myocardial cell death is the main manifestation of MI. However, all organisms have an adaptive mechanism to maintain homeostasis against hypoxia, which is mainly mediated by the hypoxia-inducible factor-1α (HIF-1α) transcription factor [4]. Under normoxia, HIF-1α is rapidly degraded and functionally inhibited through the hydroxylation of its specific proline and asparagine residues by HIF-prolyl hydroxylases (PHDs) and factor inhibiting HIF (FIH), respectively [3]. PHDs hydroxylate proline residues (Pro402 and Pro564) of HIF-1α [5], inducing von hippel–lindau protein (pVHL)-mediated ubiquitination and degradation [6]. Asparagine hydroxylation (Asn803) of HIF-1α by FIH inhibits its transcriptional activity by blocking the recruitment of p300/CBP co-activator to the C-terminal transactivation domain (CAD) [7,8,9]. Under hypoxic conditions, however, PHD and FIH activities are blocked, limiting HIF-1α hydroxylation, which results in its stabilization and activation. HIF-1α then translocates into the nucleus and forms a complex with HIF-1α, then binds to hypoxia-responsive elements (HREs), thereby upregulating the transcription of various genes involved in cell adaptation and survival under hypoxic conditions [10,11]. Recent studies have shown that HIF-1α is critical to rendering the heart resistant to hypoxic insults. For example, ischemic preconditioning provided strong cardioprotection against ischemia through the stabilization of cardiac HIF-1α, and treatment with the HIF-1α activator dimethyloxaloylglycine (DMOG) attenuated myocardial infarction [12]. In addition, HIF-1α activation by PHD2 silencing attenuated myocardial ischemia-reperfusion injury and enhanced the survival of stem cells transplanted in infarcted myocardium [13,14]. 

Low-density lipoprotein receptor-related protein 5 (LRP5) is a single-pass transmembrane protein belonging to the low-density lipoprotein receptor (LDLR) family that functions as an essential co-receptor for Wnt/β-catenin signaling activation [15,16]. As a Wnt co-receptor, loss-of-function mutations or altered expression of LRP5 have been implicated in many complex human diseases including osteoporosis [17], tumorigenesis [18,19], and hypercholesterolemia [20] through Wnt signaling abnormalities. Although the inhibition of Wnt/β-catenin signaling has been reported to protect against myocardial death caused by ischemia/reperfusion (I/R) injury [21,22,23,24], the underlying mechanisms have not yet been identified, and the role of LRP5, if any, remains unknown. Recent studies demonstrated that double deletion of LRP5 and LRP6, a sister isoform of LRP5, promotes ischemia-induced DNA damage in vivo [25], and LRP6^+/−^ mice show larger strokes in brain ischemic injury [26]. Another study reported adverse myocardial remodeling in LRP5^−/−^ mice after acute MI via abrogated Wnt signaling [27]. Genetic studies have shown that LRP5 is not optimal for β-catenin/T-cell factor (TCF) signaling stimulation [28], but that LRP6 plays a more dominant role in Wnt signaling transduction [29]. This suggests that LRP5 structure and function may be optimized to perform a different function regardless of Wnt-related signal pathways. Indeed, it was reported that LRP5 controls glucose uptake and growth of mammary epithelial cells independently from Wnt signaling, whereas LRP6 has little impact on this effect [30]. Moreover, LRP6 deficiency in the heart disrupts gap junction formation through the modulation of connexin 43 (Cx43), while LRP5 does not contribute to the regulation of the Cx43 gap junction [31]. These results further support the notion that LRP5 may have a unique role in pathophysiological conditions, especially in the ischemic myocardium. 

Therefore, the aim of this study was to examine the distinct role of LRP5 from that of LRP6 in hypoxia-induced cardiomyocytes. Toward this end, we first examined the altered levels of LRP5 and LRP6 in hypoxia-induced cardiomyocytes and determined the effects of LRP5 and LRP6 on the viability of hypoxic cardiomyocytes. Furthermore, we performed RNA-sequencing (RNA-seq) to investigate the pathways affected by LRP5 in hypoxic cardiomyocytes. Our data showed that the upregulation of LRP5 enhanced HIF-1α degradation in hypoxia-injured cardiomyocytes through its direct interaction with PHD2. We also found that the effect on the stability of HIF-1α is mediated by the phosphorylation of LRP5, which occurs independently of Wnt/β-catenin signaling. This study provides a novel target for the protection of cardiomyocyte ischemic death and improvement of heart remodeling.

## 2. Results

### 2.1. Differential Effects of LRP5 or LRP6 on Hypoxia-Induced Cardiomyocyte Death

Although LRP5 and LRP6 are structurally similar and serve as co-receptors for Wnt signal transduction, they display distinct functions according to tissue distribution and pathophysiological conditions in several reports [30,31,32,33]. To better define the role of LRP5 and LRP6 in the ischemic myocardium, we first investigated the expression levels of LRP5 and LRP6 in hypoxia-induced cardiomyocytes. 

**Figure 1:**
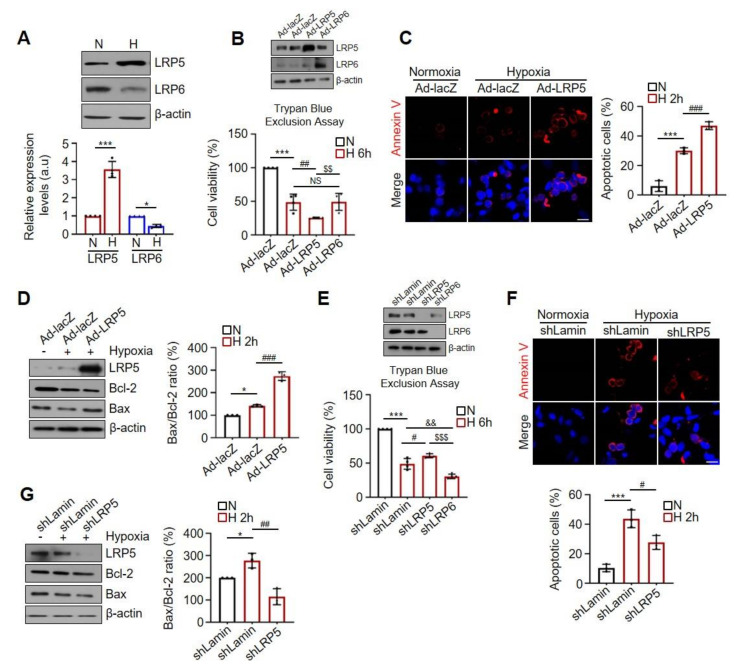
Distinct role of LRP5 and LRP6 in hypoxia-induced cardiomyocyte death. (**A**) Representative western blots showing LRP5 and LRP6 expression in cardiomyocytes exposed to hypoxic conditions (O_2_ 1.2%) for 6 h. Protein levels were normalized to β-actin levels. *n* = 4/group. Means ± SD. * *p* < 0.05, *** *p* < 0.001 versus each control. (**B**) Relative percent cell viability was assessed using trypan blue exclusion assay in Ad-lacZ-, Ad-LRP5-, and Ad-LRP6-infected hypoxic cardiomyocytes. Normoxia (O_2_ 21%) is indicated as “N”, and hypoxia is indicated as “H”. Cell viability was normalized to the normoxic Ad-lacZ control. *n* = 4/group. Means ± SD. *** *p* < 0.001 versus Normoxia; ^##^ *p* < 0.01 Ad-LRP5 versus Ad-lacZ; ^$$^ *p* < 0.01 Ad-LRP6 versus Ad-LRP5; NS, no significance. (**C**) Representative fluorescence image of Annexin V-Cy3.18 staining (red) for Ad-lacZ- and Ad-LRP5-infected hypoxic cardiomyocytes. Nuclei were stained with DAPI (blue). Scale bar, 200 μm. Apoptosis rate was quantified with SIBIA software. *n* = 3/group. Means ± SD. *** *p* < 0.001 versus Normoxia; ^###^ *p* < 0.001 Ad-LRP5 versus Ad-lacZ. (**D**) Representative western blots showing Bcl-2 and Bax expression for Ad-lacZ- and Ad-LRP5-infected hypoxic cardiomyocytes. Results are presented as a ratio and converted into %. *n* = 3/group. Means ± SD. * *p* < 0.05 versus Normoxia; ^###^ *p* < 0.001 Ad-LRP5 versus Ad-lacZ. (**E**) Relative percent cell viability assessed using trypan blue exclusion assay in AdLamin-, shLRP5-, and shLRP6-infected hypoxic cardiomyocytes. Cell viability was normalized to the normoxic Ad-lacZ control. G; *n* = 4; H; *n* = 3. Means ± SD. *** *p* < 0.001 versus Normoxia; ^#^ *p* < 0.05 shLRP5 versus shLamin; ^$$$^ *p* < 0.001 shLRP6 versus shLRP5; ^&&^ *p* < 0.01 shLRP6 versus shLamin. (**F**) Representative fluorescence image of Annexin V-Cy3.18 staining (red) for AdLamin and shLRP5-infected hypoxic cardiomyocytes. Nuclei were stained with DAPI (blue). Scale bar, 200 μm. *n* = 3/group. Means ± SD. *** *p* < 0.001 versus Normoxia; ^#^ *p* < 0.05 shLRP5 versus shLamin. (**G**) Representative western blots showing Bcl-2 and Bax expression for AdLamin- and shLRP5-infected hypoxic cardiomyocytes. Results are presented as a ratio and were converted into %. *n* = 3/group. Means ± SD. * *p* < 0.05 versus Normoxia; ^##^ *p* < 0.01 shLRP5 versus shLamin.

We observed that the expression of LRP5 was significantly upregulated under hypoxia in a time-dependent manner, while LRP6 was downregulated (Figure 1A and Figure S1A). The hypoxic stimulus also upregulated the transcriptional levels of LRP5 (Figure S1B). Next, to investigate the role of LRP5 and LRP6 in cardiomyocytes under hypoxia, we constructed adenoviral vectors expressing Myc tagged-LRP5 (Ad-LRP5) or VSVG tagged-LRP6 (Ad-LRP6) (Figure S2A,B) or expressing an LRP5 or LRP6 targeting shRNA (shLRP5 or shLRP6) to silence the endogenous expression (Figure S2C,D). Under hypoxic conditions, the overexpression of LRP5 led to a decrease in cell viability of approximately 47.72% (for trypan blue assay) or 21.32% (for MTT assay) compared to that in the Ad-lacZ control, whereas overexpression of LRP6 had no significant effect on cell viability compared to that in Ad-lacZ-treated control cells (Figure 1B and Figure S2E). 

As shown in Figure 1C,D, LRP5 overexpression caused a significant increase in annexin-V detection and Bax/Bcl-2 ratio in hypoxic cardiomyocytes. The pro-apoptotic caspase-3 activity was also increased in LRP5-overexpressed hypoxic cardiomyocytes (Figure S2F). We examined the altered expression of apoptotic markers in hypoxia-induced cardiomyocytes at 2 h for early apoptosis and 6 h for late apoptosis. In contrast, LRP5 silencing significantly prevented hypoxia-induced cell death (Figure 1E and Figure S2G) and attenuated the expression levels of apoptotic markers, such as annexin-V, Bax/Bcl-2 ratio, and caspase-3 activity compared to shLamin control cells (Figure 1F,G and Figure S2H). However, LRP6 silencing decreased cell viability by approximately 37.53% (for trypan blue assay) or 28.62% (for MTT assay) (Figure 1E and Figure S2G) and caspase-3 activity (Figure S2H) compared to shLamin control cells. These results suggest that LRP5 and LRP6 may play different roles in hypoxic cardiomyocytes, and that hypoxia-induced LRP5 expression might contribute to myocardial death. Therefore, we focused on revealing the unique role of LRP5 in the ischemic myocardium.

### 2.2. LRP5 Regulates the Expression of HIF1-α Transcriptional Target Genes

**Figure 2:**
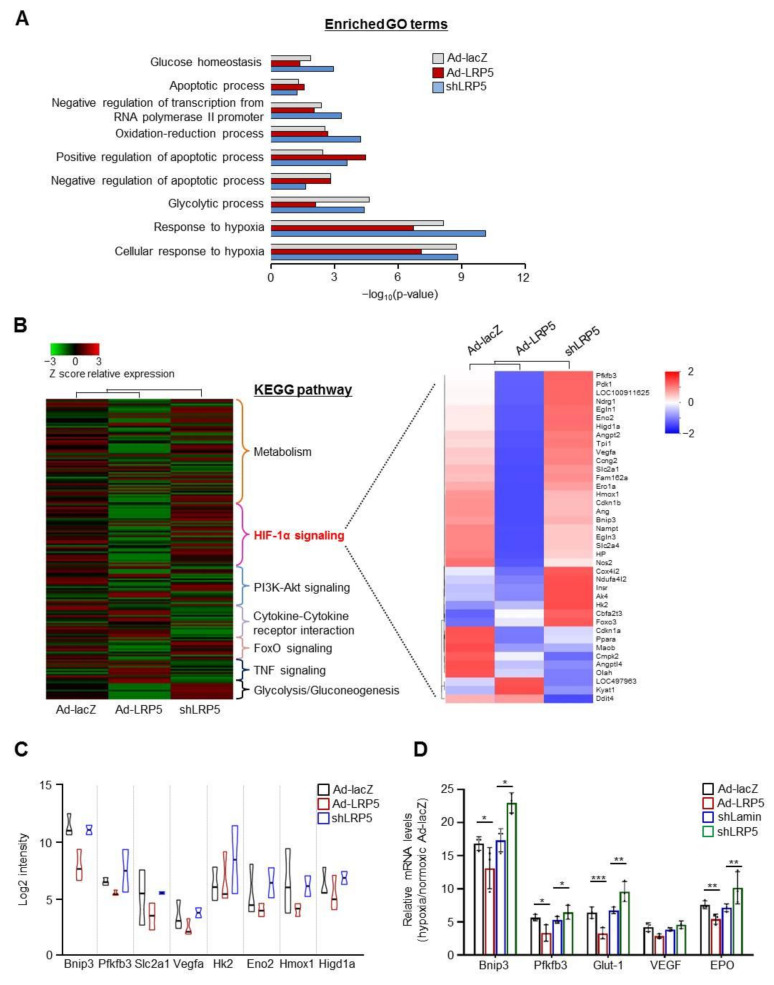
Effect of LRP5 on gene expression profile under hypoxia. (**A**) Extracted total RNA from triplicate biological samples was subjected to mRNA sequencing. GO enrichment analysis in the “Biological Process” category for genes in Ad-lacZ-, Ad-LRP5-, and shLRP5-infected hypoxic cardiomyocytes. DEGs (differentially expressed genes) were included with *p* < 0.05 and FC ≥ 2.0 vs. normoxic Ad-lacZ. (**B**) Hierarchial analysis of differentially expressed KEGG pathways (left) and a heatmap of HIF-1α transcriptional target genes (right) in the Ad-lacZ-, Ad-LRP5-, and shLRP5-infected hypoxic cardiomyocytes. Each fold change was calculated relative to the normoxic Ad-lacZ control. *p* < 0.05, FC ≥ 2.0. (**C**) The eight most significantly changed genes between Ad-lacZ and Ad-LRP5-infected hypoxic cardiomyocytes. Each fold change was calculated relative to the normoxic Ad-lacZ control. (**D**) RT-qPCR confirmation of mRNA expression of selected genes under 4 h of hypoxia. Gene expression was normalized to the arithmetic mean of three control genes (GAPDH, β-actin, and α-tubulin). Fold change was calculated relative to the normoxic Ad-lacZ control or shLamin control, *n* = 3/group. Means ± SD. * *p* < 0.05, ** *p* < 0.01, *** *p* < 0.001 versus each control.

To better understand how LRP5 promotes hypoxia-induced cardiomyocyte death and its associated cellular processes, RNA-seq was performed on LRP5-overexpressed or -silenced cardiomyocytes under hypoxia. Consistent with the enhancement of cell death by LRP5, enrichment of the gene sets in gene ontology (GO) terms related to the apoptotic process was increased, but the adaptive cellular response to hypoxia was decreased in LRP5-overexpressed cardiomyocytes. These processes were restored in LRP5-silenced cardiomyocytes (Figure 2A). Details of the GO assignment and Kyoto encyclopedia of genes and genomes (KEGG) analysis are presented in Tables S1 and S2. KEGG pathway analysis revealed that the HIF-1α signaling pathway was significantly suppressed by LRP5 overexpression and enriched by LRP5 silencing. Heat maps of genes with significant changes (≥2.0-fold change) in the HIF-1α transcriptional target gene sets (about 39 genes) that were affected by LRP5 overexpression are shown in Figure 2B. The mRNA levels of most HIF-1α transcriptional target genes was downregulated by LRP5 overexpression compared to that in Ad-lacZ-infected cells under hypoxic conditions (Figure 2C). 

To further validate the RNA-seq data, the mRNA levels of five representative HIF-1α target genes (*Bnip3l*, *Pfkfb3*, *Glut-1*, *VEGF*, and *EPO*) were analyzed using RT-qPCR. Consistent with the RNA-seq data, the mRNA levels of Bnip3l, Pfkfb3, Glut-1, and *EPO*, but not *VEGF*, were significantly downregulated by LRP5 overexpression, whereas they were markedly upregulated by LRP5 silencing (Figure 2D). These results indicate that LRP5 may modulate the transcriptional activity of HIF-1α, resulting in altered expression of its target genes in cardiomyocytes. 

All images shown are representative of those obtained from at least three independent experiments.

### 2.3. LRP5 Aggravates HIF-1α Stability in Hypoxic Cardiomyocytes

**Figure 3:**
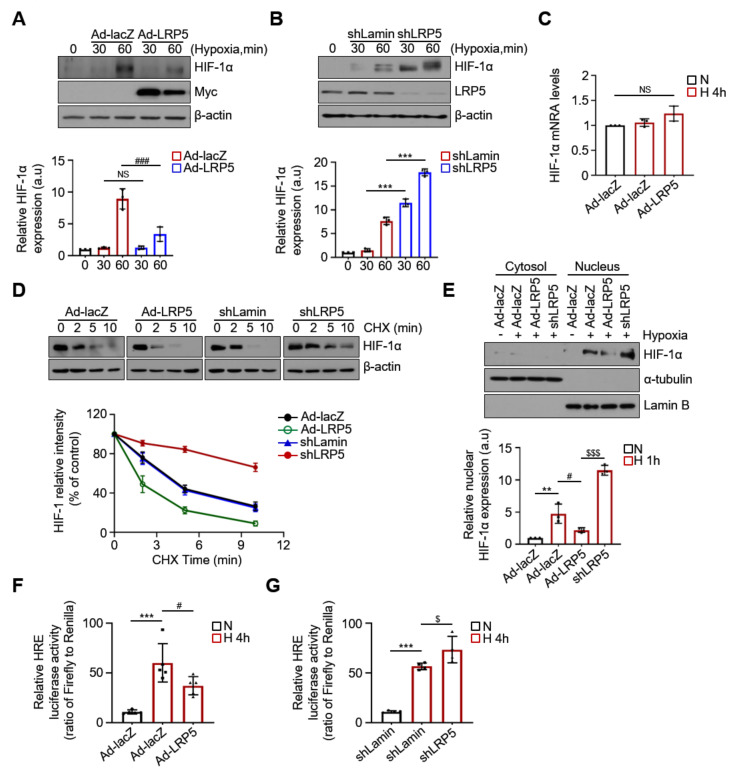
LRP5 aggravates HIF-1α stability in hypoxic cardiomyocytes. (**A**) Ad-lacZ- and Ad-LRP5-, and (**B**) shLamin- and shLRP5-infected cardiomyocytes were exposed to either normoxia or hypoxia for 30 and 60 min and harvested for western blotting. Representative western blot analysis showing HIF-1α protein levels in cardiomyocytes incubated for increasing times under hypoxia. *n* = 4/group. Means ± SD. ^###^ *p* < 0.001 Ad-LRP5 versus Ad-lacZ; NS, no significance; *** *p* < 0.001 shLRP5 versus shLamin. (**C**) HIF-1α transcript levels quantified using RT-qPCR analysis. Gene expression was normalized to the arithmetic mean of three control genes (GAPDH, β-actin, and α-tubulin). *n* = 3/group. Means ± SD. NS, no significance. (**D**) Representative half-life of the HIF-1α protein in cardiomyocytes. Ad-lacZ-, Ad-LRP5-, shLamin-, and shLRP5-infected cardiomyocytes were co-infected with Ad-HIF-1α and treated with CHX (50 μg/mL) for the indicated times and then harvested at various time points for western blotting. Protein levels were normalized to β-actin levels. *n* = 4/group. (**E**) Representative western blots showing HIF-1α expression in cytosolic and nuclear- fractionated lysates after exposure to hypoxic conditions for 60 min. α-tubulin and lamin B were used as cytoplasmic and nuclear protein controls, respectively. *n* = 4/group. Means ± SD. ** *p* < 0.01 versus Normoxia; ^#^ *p* < 0.05 Ad-LRP5 versus Ad-lacZ; ^$$$^ *p* < 0.001 shLRP5 versus Ad-LRP5. (**F**,**G**) Transactivation of HRE-luciferase activity. Cardiomyocytes were co-transfected with the HRE luciferase reporter and pRL-TK vector (control for transfection efficiency) along with (**F**) Ad-lacZ- or Ad-LRP5 (**G**) shLamin- or shLRP5- infection. Relative luciferase activity was the ratio of luciferase over renilla activity. Means ± SD. *** *p* < 0.001 versus Normoxia; ^#^ *p* < 0.05 Ad-LRP5 versus Ad-lacZ (**F**) or *** *p* < 0.001 versus Normoxia; ^$^ *p* < 0.05 shLRP5 versus shLamin (**G**). All images shown are representative of those obtained from at least three independent experiments.

We tested whether LRP5 affects HIF-1α expression under hypoxic conditions. We observed that the expression levels of HIF-1α rapidly increased under hypoxia and peaked after 1 h from induction. HIF-1α levels were markedly reduced in LRP5-overexpressed cardiomyocytes under hypoxia compared with that in Ad-lacZ-treated control cells (Figure 3A). In contrast, the expression levels of HIF-1α were enhanced in LRP5-silenced cells compared to shLamin-treated controls (Figure 3B). Since HIF-1α levels were significantly altered at 1 h under hypoxia in cardiomyocytes by the altered LRP5 levels, we hypothesized that LRP5 affects the regulation of HIF-1α stability and not the transcriptional or translational processes. HIF-1α mRNA was constitutively expressed under normoxic conditions and did not change under hypoxic conditions or LRP5 overexpression (Figure 3C and Figure S3A).

To assess whether LRP5 regulates HIF-1α stability, HIF-1α levels were evaluated in HIF-1α-overexpressed cardiomyocytes treated with cycloheximide (CHX, 50 μg/mL), a global inhibitor of protein synthesis. As shown in Figure 3D, the half-life of HIF-1α in LRP5-overexpressed cells was significantly shorter (approximately 1.8 min) than that in Ad-lacZ control cells (approximately 4 min), whereas LRP5 silencing prolonged the half-life of HIF-1α to greater than 10 min. Moreover, LRP5 silencing enhanced hypoxia-induced nuclear translocation of HIF-1α, whereas overexpression of LRP5 dramatically attenuated nuclear translocation of HIF-1α compared to control cells (Figure 3E and Figure S3B). Furthermore, HRE-dependent luciferase activity was significantly decreased in LRP5-overexpressed cardiomyocytes compared with that in Ad-lacZ-treated cells (Figure 3F), while it was increased in shLRP5-treated cardiomyocytes compared with shLamin-treated controls (Figure 3G). These results suggest that LRP5 negatively regulates HIF-1α stability, leading to a subsequent decrease in target gene transcription by HIF-1α. 

### 2.4. LRP5 Enhances Hydroxylation of HIF-1α

**Figure 4:**
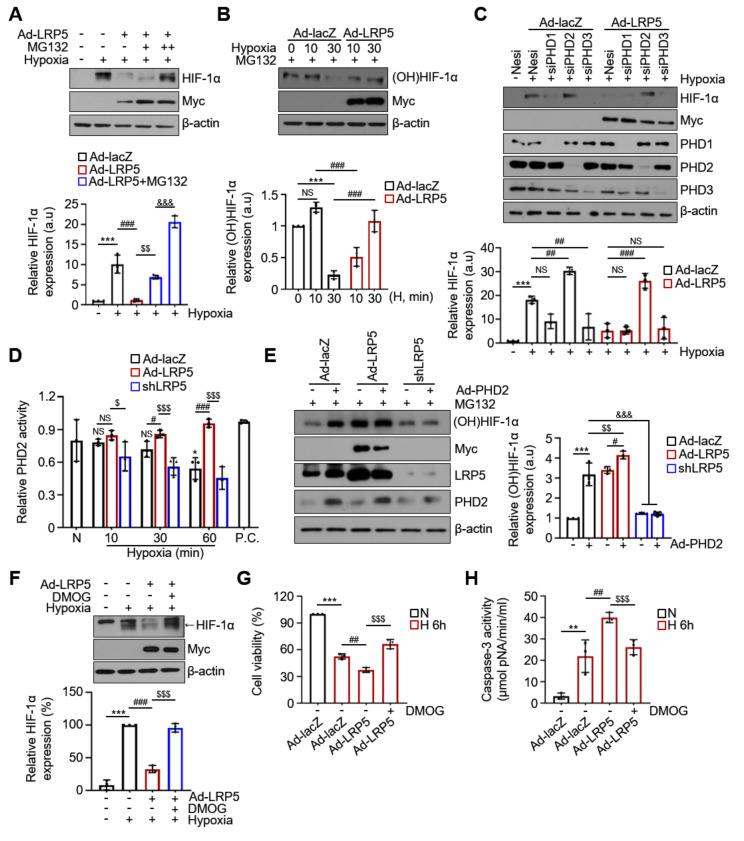
LRP5 regulates the hydroxylation of HIF-1α mediated by PHD2. (**A**) Representative western blots showing the effect of MG132 on the expression of HIF-1α in Ad-lacZ- or Ad-LRP5-infected cardiomyocytes. Cardiomyocytes were infected with the indicated adenoviruses for 48 h and treated with 1 and 10 μM MG132 for 6 h before exposure to hypoxic conditions. *n* = 4/group. Means ± SD. *** *p* < 0.001 versus Normoxia; ^###^ *p* < 0.001 Ad-LRP5 versus Ad-lacZ; ^$$^ *p* < 0.01 Ad-LRP5+MG132 (1 μM) versus Ad-LRP5 alone; ^&&&^ *p* < 0.001 Ad-LRP5+MG132 (10 μM) versus Ad-LRP5+MG132 (1 μM). (**B**) Determination of hydroxylated HIF-1α levels using a specific antibody recognizing Pro564-HIF-1α. Cardiomyocytes were infected with the indicated adenoviruses for 48 h and treated with 10 μM MG132 for 6 h before exposure to hypoxic conditions. *n* = 4/group. Means ± SD. *** *p* < 0.001 versus Normoxia; ^###^ *p* < 0.001 Ad-LRP5 versus Ad-lacZ; ^$$$^ *p* < 0.001 Ad-LRP5 versus Ad-lacZ; NS, no significance. (**C**) Representative western blot analysis showing HIF-1α protein levels. Cardiomyocytes were transiently transfected with either negative siRNA (Nesi) or PHD1-3-specific siRNAs; 24 h after transfection, cells were infected with Ad-lacZ or Ad-LRP5 for 48 h and then exposed to hypoxia for 90 min. *n* = 4/group. Means ± SD. *** *p* < 0.001 versus Normoxia; ^##^ *p* < 0.01, ^###^ *p* < 0.001 siPHDs-transfected versus Nesi; NS, no significance. (**D**) PHD2 activity was evaluated using a biotinylated HIF-1α oxygen-dependent degradation domain and hydroxylated control peptide (P.C.). *n* = 3/group. Means ± SD. * *p* < 0.05 versus Normoxia; ^#^ *p* < 0.05, ^###^ *p* < 0.001 Ad-LRP5 versus Ad-lacZ; ^$^ *p* < 0.05, ^$$$^ *p* < 0.001 shLRP5 versus Ad-LRP5; NS, no significance. (**E**) Representative western blots showing the effect of PHD2 expression on the expression of hydroxylated HIF-1α in Ad-lacZ-, Ad-LRP5-, and shLRP5-infected cardiomyocytes under hypoxic conditions (30 min). All groups were treated with 10 μM MG132 for 6 h before exposure to hypoxic conditions. *n* = 3/group. Means ± SD. *** *p* < 0.001 Ad-PHD2 versus control; ^#^ *p* < 0.05 Ad-LRP5+Ad-PHD2 versus Ad-LRP5 alone; ^$$^ *p* < 0.01 Ad-LRP5+Ad-PHD2 versus Ad-lacZ+Ad-PHD2; ^&&&^ *p* < 0.001 shLRP5 versus Ad-lacZ or Ad-LRP5 w/ or w/o Ad-PHD2. (**F**) Representative western blots showing the effect of DMOG on the expression of hydroxylated HIF-1α in Ad-LRP5-infected cardiomyocytes under hypoxic conditions (30 min). *n* = 3/group. Means ± SD. *** *p* < 0.001 versus Normoxia; ^###^ *p* < 0.001 Ad-LRP5 versus Ad-lacZ; ^$$$^ *p* < 0.001 Ad-LRP5+DMOG versus Ad-LRP5 alone. (**G**) Effect of DMOG on cell viability of Ad-LRP5-infected cardiomyocytes under hypoxic conditions. Cell viability was normalized to the normoxic Ad-lacZ control. *n* = 3/group. Means ± SD. *** *p* < 0.001 versus Normoxia; ^##^ *p* < 0.01 Ad-LRP5 versus Ad-lacZ; ^$$$^ *p* < 0.001 Ad-LRP5+DMOG versus Ad-LRP5 alone. (**H**) Measurement of caspase-3 activity in DMOG treated Ad-LRP5-infected hypoxic cardiomyocytes. *n* = 3/group. Means ± SD. ** *p* < 0.01 versus Normoxia; ^##^ *p* < 0.01 Ad-LRP5 versus Ad-lacZ; ^$$$^ *p* < 0.001 Ad-LRP5+DMOG versus Ad-LRP5 alone. All images shown are representative of those obtained from at least three independent experiments.

To further investigate how LRP5 decreases HIF-1α levels, cells were treated with the proteasome inhibitor MG132 to block the HIF-1α degradation. We observed that the level of HIF-1α was not decreased in Ad-LRP5-treated cardiomyocytes following treatment with MG132 in a dose-dependent manner under hypoxia (Figure 4A). These results demonstrate that LRP5 may regulate the proteasomal degradation of HIF-1α under hypoxic conditions. PHDs (PHD1-3) serve as cellular oxygen sensors to regulate HIF-1α stabilization by controlling its prolyl-hydroxylation (at Pro402 and Pro564) [34,35,36]. Therefore, we examined whether the hydroxylation of HIF-1α is altered by LRP5. As shown in Figure 4B, the hydroxylated levels of HIF-1α (Hyp564) were significantly suppressed (>80%) within 30 min after hypoxia induction in control cardiomyocytes. However, the levels of HIF-1α hydroxylation were not suppressed in LRP5-overexpressed cells upon hypoxia. In addition, the decreased level of HIF-1α in LRP5-overexpressed cardiomyocytes under hypoxia was significantly recovered in PHD2-silenced cells, but not in PHD1- or PHD3-silenced cells (Figure 4C).

PHD2 activity was significantly decreased under hypoxia in a time-dependent manner but increased in LRP5-overexpressed cardiomyocytes, and LRP5-silenced cells exhibited a dramatic decrease in PHD2 activity compared with Ad-LRP5 cells (Figure 4D). The hydroxylated levels of HIF-1α were significantly enhanced upon PHD2 overexpression, which was augmented by LRP5-overexpression, but dramatically suppressed by LRP5 silencing (Figure 4E). We observed that treatment with DMOG significantly restored the HIF-1α levels decreased by LRP5 under hypoxia (Figure 4F). Subsequently, cell viability was restored and caspase-3 activity was decreased by DMOG (Figure 4G,H). Together, these results demonstrate that LRP5 plays a crucial role in the regulation of PHD2-mediated HIF-1α hydroxylation.

### 2.5. LRP5 Interacts with PHD2 in Cardiomyocytes

**Figure 5:**
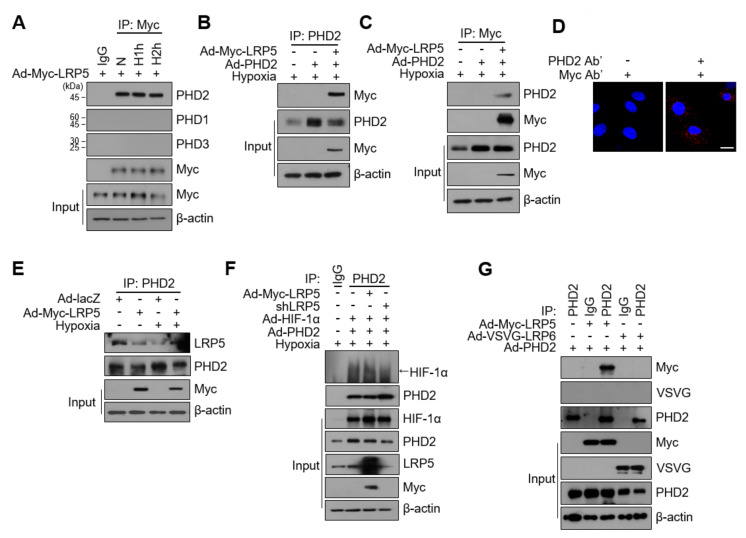
LRP5 interacts with PHD2. (**A**) Cardiomyocytes were infected with Ad-LRP5 and exposed to hypoxia as indicated. Whole-cell lysates were subjected to immunoprecipitation using an anti-Myc antibody followed by western blotting using the antibodies indicated to the right of the blot. *n* = 4/group. (**B**) Myc-tagged Ad-LRP5 and Ad-PHD2 were co-infected into cardiomyocytes and exposed to hypoxia for 60 min. Whole-cell lysates were subjected to immunoprecipitation using an anti-PHD2 antibody followed by western blotting using the anti-Myc and anti-PHD2 antibodies. *n* = 4/group. (**C**) Cardiomyocytes were prepared as in (**B**), and lysates were subjected to immunoprecipitation using an anti-Myc antibody followed by western blotting using the anti-Myc and anti-PHD2 antibodies. *n* = 4/group. (**D**) Proximity ligation assay (PLA) was performed on cardiomyocytes using anti-PHD2 and anti-Myc antibodies. Nuclei were stained with DAPI (blue). Scale bar, 200 μm. (**E**) Cardiomyocytes were infected with Ad-lacZ or Ad-LRP5, with or without hypoxia exposure. After 90 min, whole-cell lysates were subjected to immunoprecipitation using an anti-PHD2 antibody followed by western blotting using an anti-LRP5 antibody. *n* = 3/group. (**F**) Cardiomyocytes were co-transfected with adenoviruses as indicated and exposed to hypoxia. After 90 min, cell lysates were subjected to immunoprecipitation using an anti-PHD2 antibody followed by western blotting using the indicated antibodies. *n* = 3/group. (**G**) Cardiomyocytes were infected with adenoviruses as indicated, and whole-cell lysates were subjected to immunoprecipitation using an anti-PHD2 antibody or normal IgG followed by western blotting using the indicated antibodies. *n* = 3/group. All images shown are representative of those obtained from at least three independent experiments.

Given that LRP5 contributes to HIF-1α hydroxylation through the function of PHD2, we predicted that there would be functional interactions between LRP5 and PHD2. To test this hypothesis, we expressed Ad-Myc-LRP5 ectopically in cardiomyocytes, and immunoprecipitated LRP5 using an anti-Myc antibody. As shown in Figure 5A, endogenous PHD2, but not PHD1 or PHD3, was observed in the anti-Myc immunoprecipitate. Next, we co-expressed Myc-LRP5 and PHD2 exogenously and performed immunoprecipitation analyses. When cell lysates were immunoprecipitated with an anti-PHD2 antibody, Myc-LRP5 was successfully co-immunoprecipitated (Figure 5B). Reciprocally, PHD2 was co-immunoprecipitated when cell lysates were immunoprecipitated with an anti-Myc antibody (Figure 5C). Moreover, we observed an abundant PLA signal between LRP5 and PHD2 (Figure 5D). The interaction between LRP5 and PHD2 was significantly augmented in LRP5-overexpressed cardiomyocytes followed by hypoxia compared with Ad-lacZ control cells (Figure 5E).

We examined the effect of LRP5 expression on the interaction between HIF-1α and PHD2. The levels of co-immunoprecipitated HIF-1α using an anti-PHD2 antibody were consistently increased in LRP5-overexpressed cells under hypoxic conditions, as predicted. In contrast, in LRP5-silenced cells, the interaction between HIF-1α and PHD2 was significantly decreased (Figure 5F). Although LRP5 and LRP6 are homologous, LRP6 did not interact with PHD2 (Figure 5G). These results suggest that LRP5 regulates the stability of HIF-1α protein through the regulation of PHD2 activity.

### 2.6. The Regulation of HIF-1α Stability by LRP5 Is Independent of Wnt/β-catenin Signaling

**Figure 6:**
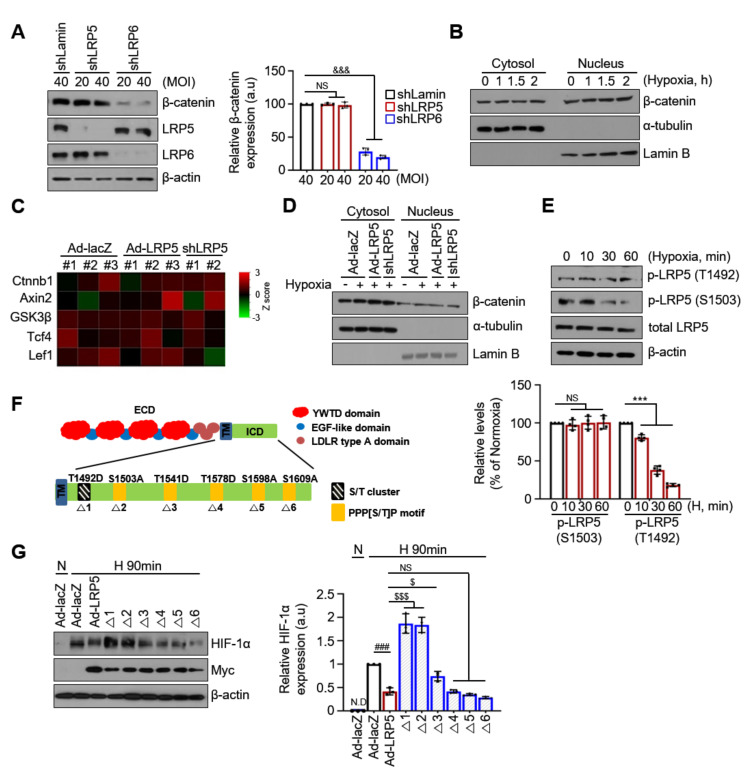
LRP5 regulates HIF-1α stability independently from the Wnt β-catenin signaling pathway. (**A**) Representative western blot analysis showing the expression of β-catenin in shLRP5 or shLRP6-infected cardiomyocytes. shLRP5/6-1: MOI 20, siLRP5/6-2: MOI 40. *n* = 3/group. Means ± SD. ^&&&^ *p* < 0.001 shLRP6 versus shLamin; NS, no significance. (**B**) Representative western blots showing β-catenin expression in cytosolic and nuclear-fractionated lysates at the indicated hypoxic times. α-tubulin and lamin B were used as cytoplasmic and nuclear protein controls, respectively. *n* = 3/group. (**C**) Heatmap of Wnt/β-catenin signaling-related genes in Ad-lacZ-, Ad-LRP5-, and shLRP5-infected hypoxic cardiomyocytes. Fold change was calculated relative to the normoxic Ad-lacZ control. Ad-lacZ and Ad-LRP5; *n* = 3, shLRP5; *n* = 2. (**D**) Representative western blots showing β-catenin expression in cytosolic and nuclear-fractionated lysates after infection with adenoviruses as indicated. α-tubulin and lamin B were used as cytoplasmic and nuclear protein controls, respectively. *n* = 3/group. (**E**) Cardiomyocytes were exposed to hypoxic conditions at the indicated times. Representative western blots showing the expression levels of p-LRP5 (S1503) and p-LRP5 (T1492). Protein levels were normalized to total LRP5 levels. *n* = 4/group. Means ± SD. *** *p* < 0.001 versus Normoxia; NS, no significance. (**F**) Schematic diagram of LRP5 phosphorylation site and site-directed mutagenesis. (**G**) Representative western blots showing the effect of LRP5 mutants on HIF-1α expression. Protein levels were normalized to β-actin levels. *n* = 3/group. N.D: non-detected. Means ± SD. ^###^ *p* < 0.001 Ad-LRP5 versus Ad-lacZ; ^$^ *p* < 0.05, ^$$$^ *p* < 0.001 versus Ad-LRP5; NS, no significance. All images shown are representative of those obtained from at least three independent experiments.

LRP5 is known as a Wnt co-receptor for the transduction of Wnt/β-catenin signaling. Phosphorylation of LRP5 by Wnt ligands inhibits glycogen synthase kinase 3β (GSK3β) phosphorylation by recruiting Axin, a scaffolding protein. Then, β-catenin is stabilized in the cytoplasm and translocated into the nucleus [15,33]. To assess whether Wnt/β-catenin signaling is involved in LRP5-mediated regulation of HIF-1α stability, we first investigated the β-catenin levels in LRP5- or LRP6-silenced cardiomyocytes. As shown in Figure 6A, LRP5 silencing did not alter β-catenin levels, whereas LRP6 silencing downregulated the levels of β-catenin. We also observed that the levels of Wnt3a were not altered LRP5 silencing (Figure S4A). β-catenin expression was not significantly changed at any time point of hypoxia either in the cytosolic or the nuclear fraction (quantitative data not shown) (Figure 6B and Figure S4B). In addition, under hypoxic conditions, the mRNA levels of Wnt/β-catenin target genes, such as *β-catenin*, *wnt3a*, and *axin2*, did not show any significant alterations (Figure 6C and Figure S4C). Furthermore, the overexpression or silencing of LRP5 did not affect β-catenin expression either in the cytoplasm or in the nucleus (quantitative data not shown) (Figure 6D). 

These results indicate that acute hypoxia does not activate Wnt/β-catenin signaling and that LRP5 does not affect Wnt/β-catenin signaling in hypoxic cardiomyocytes. Thus, the regulation of HIF-1α stability by LRP5 is independent of Wnt/β-catenin signaling. 

LRP5 has six phosphorylation sites, one S/T cluster, and five PPPSP motifs in the cytoplasmic domain that are pivotal for LRP5-participated signaling. To understand whether phosphorylation of LRP5 is involved in the regulation of PHD2-mediated HIF-1α stability under hypoxia, we investigated the phosphorylation of LRP5 and tested the effects of mutants with altered LRP5 phosphorylation sites on HIF-1α stability. We observed that exposure to hypoxia dramatically reduced the phosphorylated levels of LRP5 at Ser1503 originating from the first PPPSP motif in the intracellular domain (ICD) in a time-dependent manner, but did not alter the phosphorylated levels of LRP5 at Thr1492 (S/T cluster in ICD) (Figure 6E). Phosphorylated levels at Ser1503 were significantly reduced within 30 min of hypoxia. Given that phospho-Ser1503 was decreased within 30 min after hypoxia, and the reduction in HIF-1α stability by LRP5 occurred within 60 min (Figure 3A), we hypothesized that the altered phosphorylated LRP5 may be the signaling trigger behind the regulation of HIF-1α stability under hypoxia in cardiomyocytes. To investigate this hypothesis, we constructed constructs in which each of the six PPP(S/T)P phosphorylation motifs (T1492D, S1503A, T1541D, T1578D, S1598A, and S1609A) in LRP5 ICD were mutated (Figure 6F) and examined the effects of each construct on HIF-1α expression. Noticeably, the decrease in HIF-1α levels was prevented fully in the T1492D (△1), S1503A (△2), and partly in the T1541D (△3) mutants, but not in the T1578D (△4), S1598A (△5), and S1609A (△6) mutants (Figure 6G). The △1 and △2 mutants almost completely restored HIF-1α expression in LRP5 overexpressing cells, while △3 showed a marginal effect. These data demonstrate that the phosphorylation of some PPP[S/T]P motifs in LRP5 is involved in the regulation of HIF-1α stability in hypoxic cardiomyocytes.

### 2.7. LRP5 Silencing Protects Ischemia/Reperfusion Injury In Vivo

Considering the above results, we further analyzed the functional effect of LRP5 in myocardial injury in vivo. First, we observed that the expression of LRP5 was significantly increased in the left ventricle of LAD-ligated rat hearts (Figure S5A). Then, adenoviruses expressing lacZ, LRP5, lamin, and shLRP5 were injected into the free walls of the left ventricle of rats, and then the rats were subjected to ischemia/reperfusion injury (I/R injury) three days after injection. We observed the success of adenoviral gene delivery using X-gal staining for β-galactosidase activity in Ad-lacZ-infected heart tissues (Figure S5B). LRP5 was successfully overexpressed in the left ventricles, a finding which was confirmed by immunohistochemical staining of LRP5 using the Myc antibody (Figure S5C). Adenoviral shLRP5 successfully silenced LRP5 expression in vivo (Figure S5D).

**Figure 7:**
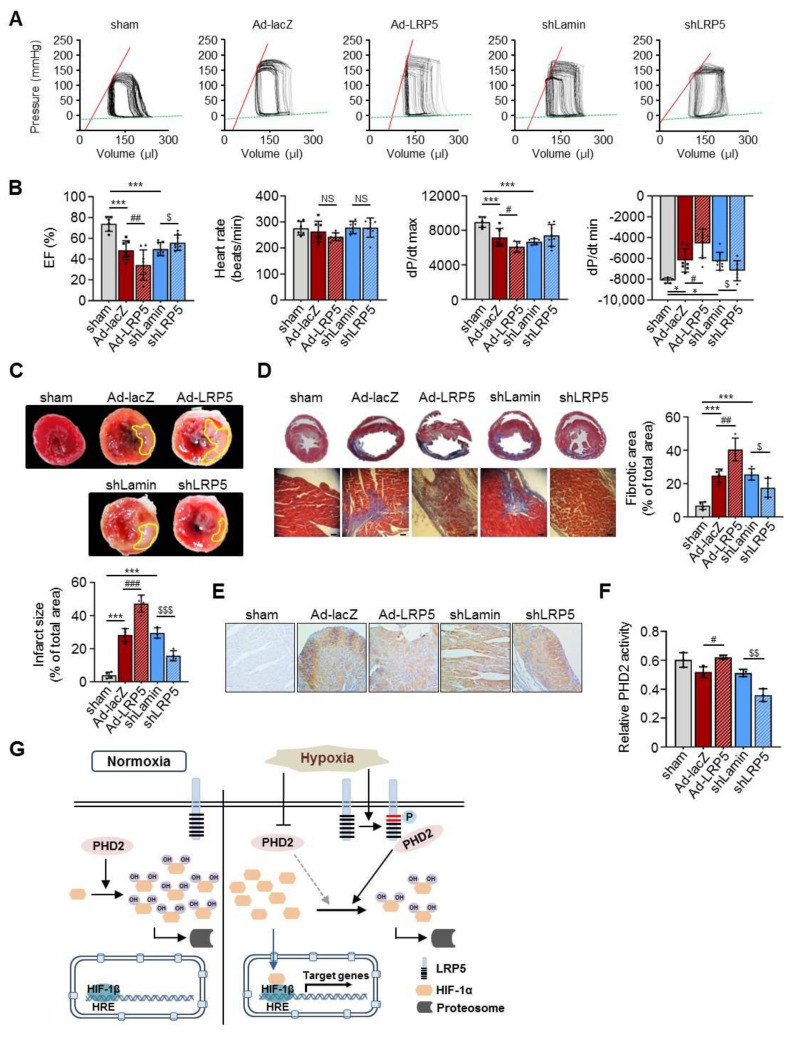
LRP5 silencing protects I/R injury in vivo. (**A**) Representative image of pressure–volume loop analysis. (**B**) Statistical analysis of the results from the hemodynamic analysis, including ejection fraction (EF), heart rate, dp/dt max, and dp/dt min in each group. *n* = 5–8 rats/group. Means ± SD. * *p* < 0.05, *** *p* < 0.001 versus sham control; ^#^ *p* < 0.05, ^##^ *p* < 0.01 Ad-LRP5 versus Ad-lacZ; ^$^ *p* < 0.05 shLRP5 versus shLamin; NS, no significance. (**C**) Representative photographs of TTC-stained myocardium sections from each experimental group. LV infarct size (white areas) is presented as a percentage of the total ventricular area. *n* = 4 rats/group. Means ± SD. *** *p* < 0.001 versus sham control; ^###^ *p* < 0.001 Ad-LRP5 versus Ad-lacZ; ^$$$^ *p* < 0.001 shLRP5 versus shLamin. (**D**) Representative Masson’s trichrome-stained images two weeks after I/R operation. Quantification is expressed as the percentage of fibrosis from the total area. Scale bars: 100 μm. *n* = 4 rats/group. Means ± SD. *** *p* < 0.001 versus sham control; ^##^ *p* < 0.01 Ad-LRP5 versus Ad-lacZ; ^$^ *p* < 0.05 shLRP5 versus shLamin. (**E**) Representative images of HIF-1α immunohistochemical staining in each experimental group. (**F**) Biotinylated HIF-1α oxygen-dependent degradation domain and hydroxylated control peptide were detected using TMB substrate at 650 nm. *n* = 4 rats/group. Means ± SD. ^#^ *p* < 0.05 Ad-LRP5 versus Ad-lacZ; ^$$^ *p* < 0.01 shLRP5 versus shLamin. (**G**) Schema of LRP5-mediated HIF-1α stability regulation under hypoxia. Under normoxia, PHD2 hydroxylates HIF-1α; then, proteasomal degradation of HIF-1α is facilitated. Under hypoxia, the hydroxylated activity of PHD2 is blocked and HIF-1α is stabilized. However, the phosphorylation of LRP5 at S1503 is increased and interacted with PHD2 under hypoxia, leading to maintenance of its hydroxylated activity. Consequently, HIF-1α is still partly degraded. All images shown are representative of those obtained from at least three independent experiments.

Hemodynamic analyses revealed that LRP5 overexpression displayed a higher end-diastolic pressure-volume relationship (EDPVR) slope than Ad-lacZ MI controls (Figure 7A). In addition, cardiac function was significantly damaged in the Ad-LRP5 MI models, as evidenced by decreased ejection fraction (EF), increased blood pressure, and a reduction in the maximal rate of decrease in the left ventricular pressure (-dp/dt) compared to Ad-lacZ MI models (Figure 7B). Conversely, LRP5-silenced models showed prevention of I/R-induced cardiac injury compared with shLamin MI models (Figure 7A,B). Furthermore, smaller infarct size and fibrosis were observed in shLRP5-MI hearts, whereas Ad-LRP5 MI showed increased infarct size and fibrosis compared with Ad-lacZ MI models (Figure 7C,D). In addition, the expression levels of HIF-1α were negatively correlated with the levels of LRP5 in the I/R-injured myocardium (Figure 7E). Moreover, we observed that PHD2 activity was significantly increased in Ad-LRP5-injected heart tissues compared with that in Ad-lacZ hearts, and the levels were rescued in shLRP5-injected hearts (Figure 7F). These results demonstrate that control of LRP5 expression enhances HIF-1α stability under ischemia, leading to decreased myocardial damage and improved heart function. 

## 3. Discussion

In this study, we show for the first time that LRP5 regulates PHD2-mediated HIF-1α stability in the ischemic myocardium. First, LRP5 expression was significantly increased in hypoxia-induced cardiomyocytes and was negatively correlated with the viability of cardiomyocytes under acute hypoxia, whereas LRP6 expression was likely positively correlated. In addition, LRP5 destabilized HIF-1α by interacting with PHD2, leading to suppression of HIF-1α transcriptional target genes. Last, the LRP5-dependent regulation of HIF-1α stability was independent of Wnt/β-catenin signaling and specifically mediated by LRP5 phosphorylation at S1503. LRP5 silencing protected against I/R injury both in vitro and in vivo. Taken together, these findings provide strong evidence suggesting the potential use of LRP5 as a therapeutic target for ischemic myocardium. 

To date, our understanding of LRP5 has been mostly limited to its role as a co-receptor in canonical Wnt signaling. Wnt signaling has diverse functions in cardiac development, homeostasis, and the repair process [33]. It is usually silent in normal adult myocardium and reactivated after experimental MI injury in various animal models, leading to cardio-hazardous results [37,38,39]. Cardiomyocyte-specific deletion of β-catenin or secreted frizzled-related proteins (sFRP) 1 and sFRP2-based Wnt antagonism has been demonstrated to improve cardiac function and reduce infarct size, fibrosis, and neutrophil infiltration [21,22,40,41,42]. In addition, injection of DKK2, an antagonist of Wnt signaling, binding to LRP5 and LRP6, was shown to be beneficial for infarct healing in a cardiac ischemia/reperfusion model [43]. These results were partly supported by our results showing that LRP5 overexpression increases I/R injury, whereas LRP5 silencing decreases it. However, the direct role of LRP5 and its underlying mechanism in ischemic myocardium have not yet been reported. 

LRP5 is structurally similar to LRP6, and these two receptors have been proposed to function similarly in the same contexts and signaling pathways. It was reported that loss of either LRP5 or LRP6 alone did not show any alterations in intestinal differentiation [44]. Another study found that LRP5 and LRP6 redundantly control skeletal development in the mouse embryo [45]. However, recent studies showed that they display distinct functions [46]. For example, although LRP5 is expressed during embryogenesis, Lrp5^−/−^ mice are viable and do not show any developmental abnormality, whereas Lrp6^−/−^ mice die at birth, indicating that LRP6 is more critical than LRP5 in developmental processes [44,47]. Moreover, LRP6 appears to be crucially important for glucose and lipid metabolism signaling [29,48], while LRP5 plays a more important role in bone-mass phenotype [47,49]. 

In this study, we identified the unique role of LRP5 in the ischemic myocardium. LRP5 overexpression significantly increased apoptotic signals and subsequently led to decreased viability of cardiomyocytes under hypoxia. We further identified for the first time the direct interaction between LRP5 and PHD2, leading to enhanced HIF-1α degradation and decreased transcriptional levels of its target genes, Pfkfb3, Glut-1, and EPO. In mammals, three PHD isoforms (termed PHD1-3) exist, and PHD2 and PHD3 are known to be highly expressed in the heart [50]. Consistently, although we observed that both PHD2 and PHD3, but not PHD1, were significantly upregulated under hypoxic conditions, LRP5 only interacted with PHD2, increasing its hydroxylation activity. All PHD enzymes operate on both HIF-1α and HIF-2α, although relative isoform selectivity is observed. PHD2 is the most important enzyme in regulating the general levels of HIF-1α, whereas the more tissue-restricted isoforms PHD1 and PHD3 seem to be somewhat more active against HIF-2α [36,50]. 

Our data from the RNA-seq analysis showed an evident relationship between LRP5 and HIF-1α signaling in cardiomyocytes. The expression of most HIF-1α transcriptional target gene sets (approximately 39 genes) was affected by LRP5 levels, with the exception of several genes. These exceptions might be due to variations in the experimental conditions such as the hypoxia incubation time. Moreover, these results are consistent with previous reports showing that HIF-1α governs the acute adaptation to hypoxia, whereas HIF-2 and HIF-3 expression is triggered during chronic hypoxia [51]. Although some reports have shown that both HIF-1α and HIF-2α were stabilized under low-oxygen conditions, HIF-1α levels remained high to mediate the acute response and decreased during prolonged hypoxia, whereas HIF-2α stabilized to regulate the cellular response under prolonged hypoxia [52,53]. HIF-2α seems to be more stable than HIF-1α at higher oxygen levels. Other reports also support that HIF-2α levels are stabilized relatively later and tend to play a crucial role during chronic hypoxia (24–72 h) [54,55,56,57]. Therefore, HIF-1α activation in acute ischemic hearts has been considered a target for therapeutic strategies. Based on our results, we suggest the potential use of LRP5 as a target to regulate HIF-1α activation in the ischemic myocardium. 

Moreover, we compiled evidence demonstrating the different functions of LRP5 and LRP6 in the ischemic myocardium. LRP5 and LRP6 showed opposite expression patterns in hypoxia-induced cardiomyocytes. The viability of cardiomyocytes under hypoxia was significantly decreased in LRP5-overexpressed cells. Consistently, apoptotic signals were more activated in hypoxic cardiomyocytes after LRP5 overexpression. In addition, it is remarkable that LRP6 deletion led to a decrease in cell viability and increases in apoptotic signals including caspase-3 activity and Bax/Bcl-2 ratio (Figure 1G–I). We further observed that LRP6 did not interact with PHD2. These results demonstrated that LRP5 and LRP6 have distinct roles in cardiac biology. A previous study showed that the double deletion of LRP5 and LRP6 promotes ischemia-induced DNA damage in vivo [25]. However, this study could not address the independent role of LRP5 because the results of the study were obtained using double deletion mutants to achieve absolute abrogation of Wnt signaling. Conversely, another study showed the protective potential of LRP6 in brain ischemic injury [26]. Therefore, the unique role of LRP6 distinguished from LRP5 in ischemic myocardium and its underlying mechanisms need to be further investigated.

Another remarkable difference between LRP5 and LRP6 function is that their role in Wnt signal activation might be different, although both LRP5 and LRP6 are known as Wnt co-receptors. Similarly, while LRP5 weakly activates Wnt signaling in mammalian cell cultures in the absence of exogenous Wnt ligands, LRP6 is highly active in this regard. The difference between the two receptors might be due to the differential signaling activity in the cytoplasmic domain [16,28,44]. In addition, we showed that the regulation of HIF-1α stability by LRP5 is mediated by the phosphorylation of PPP[S/T]P motifs, specifically T1492 and S1503, in the cytoplasmic domain. These results indicate that PPPSP motifs in LRP5 or LRP6 independently participate in their unique signaling pathways. Consistently, our results also demonstrated that only LRP6 silencing in cardiomyocytes was implicated in the downregulation of β-catenin expression (Figure 6A), indicating that LRP6 is a more effective transducer of Wnt activation than LRP5. As shown in Figure 6B, β-catenin levels did not change at low O_2_ levels (1.2%) and short exposure time (30–120 min), indicating that the Wnt/β-catenin signaling pathway is not affected by acute hypoxic conditions. Several studies have reported that Wnt signaling is activated during remodeling processes such as wound healing followed by MI and that the increase in Wnt signaling-related genes is observed one week after MI, gradually decreasing after two weeks [58,59]. Therefore, the role of LRP5 in the myocardium under acute ischemia is independent of Wnt signaling, while Wnt signaling activation occurs during infarct healing in inflammation, fibrosis, and neovascularization.

In this study, we demonstrate a novel regulatory mechanism in ischemic myocardium consisting of the direct interaction between LRP5 and PHD2, which influences the stability of HIF-1α, a key molecule involved in the protection of cardiac death under ischemia. Indeed, hydroxylated HIF-1α expression was significantly increased by overexpression of PHD2 alone. Overexpression of LRP5 alone also resulted in a similar trend of increased hydroxylated HIF-1α expression. However, siRNA knockdown of LRP5 and overexpression of PHD2 did not further upregulate the expression of hydroxylated HIF-1α (Figure 4E). In addition, our findings indicate that the phosphorylation of LRP5 at S1503 is increased, thereby resulting in the interaction with PHD2 and maintaining its hydroxylation activity, then promoting the proteasomal degradation of HIF-1α (Figure 7G). 

These findings suggest a novel role of LRP5 in the regulation of HIF-1α under hypoxia and that LRP5 might be a fine regulator that controls HIF-1α stability depending on the oxygen concentrations under hypoxic conditions. Although the PHD inhibitors are licensed for clinical use in chronic kidney disease [60], our data provide a new cardiomyocyte-or heart-specific target against hypoxic insult. This may expand the research approach for developing a treatment for ischemia-induced heart injury. We suggest that the role of LRP5 as a regulator of HIF-1α stability under hypoxic conditions can be exploited as a novel therapeutic strategy targeting LRP5 for the treatment of myocardial infarction. 

## 4. Materials and Methods

### 4.1. Primary Culture of Rat Cardiomyocytes

Neonatal rat cardiomyocytes were isolated from the hearts of rat pups using the method previously described [61]. Briefly, isolated hearts were washed with Dulbecco’s phosphate-buffered saline solution (pH 7.4, WELGENE, Gyeongsan-si, Korea), minced to approximately 0.5 mm^3^ sized pieces, and treated with 5 mL collagenase II (1.4 mg/mL, 270 units/mg, Gibco; Thermo Fisher Scientific, Waltham, MA, USA) solution for 5 min. Then, the supernatants were removed and washed with alpha modification minimum essential medium (α-MEM) with 10% fetal bovine serum (FBS) and 1% penicillin/streptomycin solution (Carpricorn Scientific, Ebsdorfergrund, Germany). The pellets were resuspended in collagenase II and incubated in a 37 °C humidified atmosphere chamber containing 5% CO_2_ for 7 min. This procedure was repeated until the tissues were completely digested. The resulting cell pellets were resuspended in α-MEM, and cells were attached to 100 mm culture dishes for 30 min at 37 °C. Non-adherent cardiomyocytes were cultured for the experiment in α-MEM containing 10% FBS, 1% penicillin/streptomycin solution, and 0.1 mM Bromodeoxyuridine (BrdU, Sigma–Aldrich, St. Louis, MO, USA) to eliminate fibroblast expansion.

### 4.2. Production, Purification, and Administration of the Adenoviruses

Recombinant adenoviruses expressing full-length human LRP5 with a C-terminal Myc tag (named Ad-LRP5) and full-length human LRP6 with a C-terminal VSVG tag (named Ad-LRP6) were constructed using ViraPower™ Adenovirus Expression System (Invitrogen; Thermo Fisher Scientific). An adenoviral vector expressing lacZ-β-galactosidase (named Ad-lacZ) was used as infection control. The constructs were then used in an LR recombination reaction with the pAd/CMV/V5/DEST gateway vector to generate an adenoviral expression clone. Then, the constructed adenoviral vector was transfected into 293A cells using Lipofectamine 2000 reagent (Invitrogen; Thermo Fisher Scientific) after digestion with Pac I restriction enzyme. Adenoviral particles were titered using the Adeno-X™ qPCR titration kit (Takara; Clontech, Otsu, Japan). Isolated cardiomyocytes were infected with Ad-LRP5 (multiplicity of infection (MOI) of 10) or Ad-LRP6 (MOI of 10) for 48 h before exposure to hypoxic conditions. The shRNA targeting rat LRP5 (named shLRP5) and rat LRP6 (named shLRP6) were generated using a BLOCK-iT™ Adenoviral RNAi Expression system (Invitrogen; Thermo Fisher Scientific). The RNAi nucleotide sequence targeting rat LRP5 was 5′-GCUGUUCAGCCAG-AAAUUU-3′ and that targeting rat LRP6 was 5′-GGUUGUUCCCAUUUGUGUU-3′. Double-stranded oligonucleotides cloned in the BLOCK-iT™ U6 entry vector were used in an LR recombination reaction with the pAd/BLOCK-iT™-DEST vector to generate an adenoviral expression clone. The pAd-GW/U6-laminshRNA adenoviral vector (named shLamin) was used as an infection control. Adenoviral particles were titered using the Adeno-X™ qPCR titration kit (Takara; Clontech). Isolated cardiomyocytes were infected with shLRP5 (MOI of 40) or shLRP6 (MOI of 40) for 72 h before exposure to hypoxic conditions. Point mutagenesis of the LRP5 phosphorylation site was performed with a QuikChange site-directed mutagenesis Kit (Agilent Technologies, Santa Clara, CA, USA) according to the manufacturer’s instructions. The lists of mutagenic primers are provided in Table 1. 

### 4.3. siRNA Transfection

For siRNA-mediated knockdown of PHD1-3, cells were transfected with 60 nM of the targeting or negative siRNA using siLentFect (Bio-Rad Laboratories, Hercules, CA, USA) for 72 h according to the manufacturer’s instructions. The sequences of siRNA targeting PHD1-3 and the negative control are provided in Table 1. 

### 4.4. Hypoxia Treatment

Hypoxia treatment was induced using the method previously described [62,63]. Briefly, cardiomyocytes were carefully washed twice with hypoxic medium (serum-free and replacing with N_2_ gas for 30 min) and subjected to hypoxia to mimic the in vivo conditions of myocardial ischemia. The cells were placed in an incubator at 37 °C. N_2_ (95%) and CO_2_ (5%) were flushed into the incubator to bring the oxygen content down to 1.2%, which was monitored using an oxygen probe (Vision Scientific, Korea). Normoxic cardiomyocytes were maintained at 37 °C in a separate incubator with a normoxic atmosphere (O_2_ 21%).

### 4.5. Cell Viability

For the cell viability assay, experimental cells were evaluated on both MTT and Trypan blue assays. MTT assay was performed using the CellTiter 96 Aqueous One Solution Cell Proliferation Assay (Promega, Madison, WI, USA) according to the manufacturer’s instructions. The absorbance was measured at 490 nm using an ELISA reader (TECAN, infinite M200 PRO; Life Sciences, Redmond, WA, USA). For Trypan blue assay, cells were detached by trypsinization and stained with 0.4% Trypan blue dye. Viable cells were counted under the microscope using a hemocytometer counter. The viability of the control was regarded as 100%.

### 4.6. Luciferase Reporter Assay

To analyze the in vitro relationship between LRP5 and HIF-1α under hypoxic conditions, a luciferase reporter assay was performed. A luciferase reporter construct containing three HREs was purchased from Addgene (26731). HRE-luciferase reporter plasmid and Renilla luciferase vector (pRL-TK; Promega) were transiently transfected into control, LRP5-overexpressed- or LRP5-silenced cardiomyocytes for 24 h. Next, cells were exposed to normoxic or hypoxic conditions. Cell lysates were harvested for both firefly and Renilla luciferase assay analyses using the Dual-Luciferase Reporter Assay System (Promega) as described in the manufacturer’s protocol. The HRE-Luciferase activity was measured using a Micro-Lumat Plus LB96V luminometer (EG&G Berthold, Bad Willdbad, Germany). Data were normalized to the Renilla luciferase activity to account for differences in transfection activity.

### 4.7. Nuclear Extract Preparation

Cells were lysed with cytosol extraction buffer (10 mM HEPES; pH 7.5, 3 mM MgCl_2_, 14 mM KCl, 5% glycerol, and 1 mM DTT) for 10 min on ice. Then, NP-40 at a 0.4% final concentration was added and vortexed for 10 s. The cell suspensions were centrifuged for 2 min at 5000 rpm at 4 °C, and the supernatants containing the cytosolic fractions were removed. The pellets were washed three times in the same lysis buffer and then lysed with nuclear extraction buffer (10 mM HEPES; pH 7.5, 3 mM MgCl_2_, 400 mM NaCl, 5% glycerol, and 1 mM DTT) and sonicated. After 30 min of incubation, the pellet suspensions were centrifuged for 10 min at 14,000 rpm at 4 °C. Supernatants containing the nuclear fractions were used for western blotting. Anti-lamin B (sc-374015; Santa Cruz Biotechnology) and anti-α-tubulin (sc-5286; Santa Cruz Biotechnology) were used as nuclear and cytoplasmic loading controls, respectively.

### 4.8. Apoptosis Assay

The hypoxia-induced cardiomyocyte apoptosis rate was determined using the Annexin V-Cy3 apoptosis detection kit (Sigma–Aldrich) or colorimetric Caspase 3 Assay kit, (Sigma–Aldrich) according to the manufacturer’s instructions. Annexin V-Cy3 labeled cells were then visualized using a laser scanning confocal microscope (Fluoview FV1000, Olympus, Tokyo, Japan). For the detection of Caspase-3 activity, cell lysates were combined with an equal amount of substrate reaction buffer containing caspase-3 colorimetric substrate (Ac-DEVD-pNA). Then, the absorbance was measured at 405 nm using an ELISA reader (TECAN, infinite M200 PRO; Life Sciences). 

### 4.9. PHD2 Activity Assay

To analyze the in vitro activity of PHD2, we used biotinylated HIF-1α oxygen-dependent degradation domain (Biotin-DLDLEALAPYIPADDDFQL; from amino acids 556 to 575 of HIF-1α) and hydroxylated control (Biotin-DLDLEALAP[OH]YIPADDDFQL) peptides immobilized on streptavidin and bovine serum albumin (BSA)-coated 96 well plates. After exposure to hypoxia, cardiomyocytes were harvested and lysed with hypotonic buffer (20 mM HEPES; pH 7.4, 5 mM NaF, 10 μM Na_3_VO_4_, 0.1 mM EDTA, protease inhibitor cocktail, and 2 mM DTT) for 20 min. Then, NP-40 at a 0.5% final concentration was added. Equal quantities of protein (50 μg/well) were incubated with reaction buffer (Tris-Cl; pH 7.5, 5 mM KCl, 1.5 mM MgCl2, 2 mM DTT, 0.5 mM 2-OG, and 1 mM ascorbate) for 1 h at RT. Samples were incubated with anti-hydroxy-HIF-1α primary antibody (3434P; Cell Signaling Technology), followed by the addition of goat anti-rabbit HRP-conjugated secondary antibody. Peptide hydroxylation was detected at 650 nm using TMB substrate. 

### 4.10. Proximity Ligation Assay

In situ interaction between LRP5 and PHD2 was detected using a Proximity Ligation Assay kit, Duolink (Sigma–Aldrich, DUO92101). Cells were fixed and permeabilized as described in the immunofluorescence method. Antibody incubation and probe amplification were performed according to the manufacturer’s instructions. A negative control experiment was performed where only one primary antibody was incubated with the PLA probes. The fluorescent signals in the cells were visualized using laser scanning confocal microscopy (Fluoview FV1000, Olympus). 

### 4.11. Immunoprecipitation Assay and Western Blotting

To analyze the interaction between LRP5 and PHD2, we performed an immunoprecipitation assay. Cells were washed once in PBS and lysed in RIPA buffer containing protease inhibitor cocktail and 1 mM phenylmethylsulfonyl fluoride (PMSF). Protein concentrations were determined using a Bradford Protein Assay Kit (Bio-Rad). Equal quantities of protein were separated on 6–12% SDS-PAGE and transferred to a polyvinylidene difluoride membrane (Bio-Rad). After membrane blocking with Tris-buffered saline-Tween 30 (TBS-T, 0.1% Tween 20) containing 5% skim milk for 1 h at RT, the membranes were incubated with primary antibodies overnight at 4 °C using the following antibodies: rabbit anti-HIF-1α (NB100-479), rabbit anti-PHD2 (NB100-2219), rabbit anti-PHD3 (NB100-139) from Novus Biologicals; rabbit anti-LRP5 (5731), rabbit anti-LRP6 (2560), rabbit anti-Hydroxy-HIF (Pro564) (3434P), rabbit anti-Bax (2772), mouse anti-Myc tag (2276) and rabbit-anti LRP6 (phospho S1490) (2568) from Cell Signaling Technology; rabbit anti-β-catenin (ab32572), rabbit anti-PHD1 (ab113077) and rabbit anti-LRP5 (phospho T1492) (ab203306) from Abcam; mouse anti-Bcl-2 (610538) from BD Biosciences; rabbit anti-VSVG tag (ADI-MSA-115) from Enzo Life Sciences. Blots were washed five times for 25 min with TBS-T (0.1% Tween 20) and then incubated with a horseradish peroxidase-conjugated secondary antibody for 1 h at RT. Immuno-reactive proteins were detected using an ECL system. Image J software was used for quantification. For the immunoprecipitation assay, lysates were precleared with protein A/G-Agarose beads (Santa Cruz Biotechnology) before adding the antibody. Then, after removing the protein beads by centrifugation, the supernatants were incubated overnight at 4 °C with the following primary antibodies: mouse anti-myc tag (2276) from Cell Signaling Technology, mouse anti-PHD2 (AM09354PU) from Origene Technologies or isotype control immunoglobulin G (IgG) (sc-2025) from Santa Cruz Biotechnology. Next, A/G-Agarose beads were added and incubated for 4 h at 4 °C with rotation, after which bead-antibody complexes were washed four times with EBC washing buffer (20 mM Tris, 500 mM NaCl, 1 mM EDTA, 0.5% NP-40). Immunoprecipitated proteins were denatured in SDS sample buffer and boiled for 5 min. Samples were analyzed using western blotting. 

### 4.12. Gene Expression Analysis and Heat Map

Total RNA isolation from Ad-lacZ-infected normoxic cells and Ad-lacZ- or Ad-LRP5-infected hypoxic cells was performed using Trizol reagent (QIAGEN, Hilden, Germany) according to the instructions provided by the manufacturer. mRNA sequencing and analysis were performed by the e-biogen Company. A *p*-value of < 0.05 was used to generate lists of differentially expressed genes. Gene Ontology enrichment analysis and KEGG enrichment analysis were conducted using the DAVID 6.8 tool (http://david.abcc.ncifcrf.gov/, accessed date: 20 February 2020). Heat maps were created using the FunRich 3.1.3 software (http://www.funrich.org/, accessed date: 20 February 2020). A *p*-value of < 0.05 was regarded as significant.

### 4.13. Quantitative Real-Time PCR (RT-qPCR) Analysis 

Total RNA was isolated from cells using Trizol reagent (QIAGEN) according to the instructions provided by the manufacturer. Total RNA was subjected to reverse transcription using the 1st-Strand cDNA Synthesis kit (Takara). Real-time quantitative PCR with realHelix TM qPCR kit (NanoHelix, Daejeon, Korea) was performed by the SYBR Green method using an Applied Rotor-Gene 3000^TM^ (Corbett Research, Sydney, Australia). Primer efficiency was calculated for RT-qPCR using the slope of the calibration curve according to the equation: E = 10^[−1/slope]^ [64]. Relative mRNA levels were normalized to the arithmetic mean of values from three reference genes (GAPDH, β-actin, and α-tubulin). A list of the primers is provided in Table 1. 

### 4.14. Immunofluorescence Staining

Cardiomyocytes were cultured in 24-well plates with cover glass. Cells were fixed with 4% paraformaldehyde for 15 min and permeabilized with 0.2% Triton X-100 for 10 min at RT. After washing, cells were blocked with 2% BSA in PBS for 1 h, removed from the blocking solution, and incubated overnight at 4 °C with rabbit anti-HIF-1α (NB100-479; Novus biologicals) and mouse anti-LRP5 (sc-390267; Santa Cruz Biotechnology). Cells were washed and incubated with mouse anti-rabbit IgG-TR (sc-3917; Santa Cruz Biotechnology) and goat anti-mouse IgG-FITC (ab6785; Abcam) at RT for 1 h in the dark. Then, cells were gently washed with PBS and visualized using laser scanning confocal microscopy (Fluoview FV1000, Olympus).

### 4.15. Animal Models of Ischemia/Reperfusion Injury

All animal studies were conducted in accordance with the International Guide for the Care and Use of Laboratory Animals. The protocol was approved by the Animal Research Committee of the Chosun University School of Medicine (Protocol No. CIACUC2018-S0004). Sprague Dawley Rats (seven weeks old) and neonatal rats on Sprague Dawley background were purchased from Samtako Bio Korea company under specific pathogen-free (SPF) conditions. Animals were fed a standard laboratory diet with water and kept on a 12-h light/12-h dark cycle in a temperature-controlled room. An ischemic/reperfusion (I/R) model was established in 7-week-old Sprague-Dawley male rats (250 g) by surgical occlusion of the left anterior descending (LAD) coronary artery as described previously [63,65]. Briefly, rats were anesthetized with a Zoletil 50 (Tiletamine 25 mg, Zolazepam 25 mg/mL)-Rompun (Xylazine HCL 23.3 mg/mL) mixture (5:1, 45 mg/kg intraperitoneally) and ventilated with room air via tracheal intubations connected to a Harvard ventilator. Following the left lateral thoracotomy located between the third and fourth ribs, the pericardium was incised and the left anterior descending coronary artery was ligated with sterile 6-0 silk suture. Proper ligation for the myocardial infarction model was verified by visual observation of the left ventricle wall turning pale. After 60 min of regional myocardial ischemia, the ligation was released after removal of the 6-0 silk suture to allow reperfusion. Sham-operated animals underwent the same procedure without occlusion of the left anterior descending coronary artery. To determine the effect of LRP5 in myocardial infarction models in vivo, animals were injected with adenoviral constructs expressing lacZ, LRP5, shLamin, or shLRP5 (2 × 10^11^ particles) before operating. The adenovirus constructs were injected at three different sites into the anterior left ventricular free wall using a 32G needle. Sham-animals were injected with saline. Adenovirus infection efficiency was assessed using western blot analysis after three days. Sham, Ad-lacZ, Ad-LRP5, shLamin, and shLRP5 injected experimental animals were re-anesthetized three days later, and left anterior descending coronary artery ligation was performed.

### 4.16. X-gal Staining of Tissues

To analyze the in vivo expression of adenoviral vectors, we performed a β-galactosidase activity assay. For visualization of β-galactosidase activity, lacZ adenovirus-injected fresh rat heart tissues were excised and post-fixed with 4% paraformaldehyde for 4 h at 4 °C followed by incubation with 30% sucrose overnight at 4 °C. The heart tissues were mounted on a standard cryomold with OCT compound and cut into 20 μm serial sections using a cryostat (Thermo Fisher Scientific). Then, serial sections were stained overnight at 37 °C with X-gal using a senescence β-galactosidase histochemical staining kit (Sigma-Aldrich). Stained sections were captured using microscopy (Olympus). 

### 4.17. Histological Analysis and Immunohistochemistry

To analyze the functional effects of adenoviral vector introduction in vivo, we performed histological analysis and immunohistochemistry. Briefly, the hearts were excised and fixed with 4% formaldehyde. After dehydration, the heart tissues were embedded in paraffin wax and sectioned at 4 μm thickness. The levels of collagen deposition were determined with Masson’s trichrome staining (American MasterTech, Lodi, CA, USA). Areas of fibrosis tissue and total tissue in each field were measured and evaluated as the percentage of fibrosis tissue area to total tissue area using the SABIA (Solution for Automatic Bio-Image Analysis) program (e-Biogen, Seoul, Korea). In addition, immunohistochemistry was performed according to the avidin biotinylated-horse radish peroxidase (HRP) complex (ABC)-based method using the Vectastain ABC kit (Vector Laboratories, Burlingame, CA, USA). Briefly, paraffin-embedded heart sections were deparaffinized and rehydrated through xylenes and graded ethanol series before antigen retrieval. The sections were then incubated with 0.3% hydrogen peroxide in 60% methanol for 30 min and blocked with normal goat serum for 1 h at 4 °C. This was followed by incubation with primary antibodies. Immunoreactivity was achieved using 3′-diaminobenzidine (DAB, Sigma–Aldrich). The sections were counterstained with Mayer’s hematoxylin solution (Sigma–Aldrich), dehydrated, defatted, and mounted with malinol (Muto Pure Chemicals, Tokyo, Japan). The stained structures were observed under a microscope (BX41, Olympus). Image J software was used for quantification. Five images from non-overlapping fields of stained sections were captured from each section per animal using microscopy (Olympus), and quantitative analysis of positive staining areas (%) in images was done using Image J software. 

### 4.18. Infarct Size Determination

Sham and ischemic/reperfusion (I/R)-injured animals were euthanized with CO_2_ two weeks after the operation, and their hearts were excised and washed with PBS. The heart was snap-frozen in liquid nitrogen, sectioned transversely, and incubated in 1% 2,3,5-Triphenyltetrazolium chloride (TTC) solution (pH 7.4 buffer at 37 °C) for 20 min. The tissue was immersed in 10% PBS buffered formalin overnight at 4 °C. The size of myocardial infarction was evaluated as a percentage of the sectional area of the infarcted tissue of the left ventricle to the sectional area of a total ventricular area. Overall infarct size was assessed using Image J software (National Institutes of Health, Bethesda, MD, USA).

### 4.19. Hemodynamic Analysis

The hemodynamic assessment was performed using a Mikro-Tip Research Pressure System (Millar Instruments, Houston, TX, USA). Briefly, rats were anesthetized, and 2F Pressure-volume (P-V) catheter (SPR-838, Millar Instruments) was inserted into the right carotid artery and advanced into the ascending aorta. Then, the catheter was advanced into the LV cavity until stable P-V loops were obtained. EF, Heart rate, dP/dt max, and dP/dt min were computed and calculated using PV analysis program (PVAN, Millar Instruments). The slopes (Ees) of the LV end-systolic P-V relationship (ESPVR) were calculated as load-independent indices of LV contractility. At the end of each experiment, 100 μL of hypertonic saline was injected intravenously, and from the shift of P-V relations, parallel conductance volume was calculated by the software and used for correction of the cardiac mass volume. Finally, the data were analyzed using LabChart data analysis software (Millar Instruments).

### 4.20. Statistical Analysis

All quantified data from at least triplicate samples were analyzed with GraphPad Prism 8.0 software (La Jolla, CA, USA). Data are expressed as means ± SD. Statistical comparisons between two groups were performed using Student’s *t*-test. Statistical comparisons among multiple groups were performed using one-way ANOVA followed by the Bonferroni post hoc test when the F statistic was significant. A two-tailed *p* < 0.05 value was considered statistically significant.

## Figures and Tables

**Table 1:** List of primers and oligos used for qPCR, site-directed mutagenesis, and siRNA silencing.

qPCR Primers		
Gene	Forward (5′–3′)	Reverse (5′-3′)
LRP5	AGGCCCTACATCATTCGAGG	GGGGTCTGAGTCCGAATTCA
BNIP3	CTGCACTTCAGCAATGGG	CTCTTGGAGCTGCTTCGT
PFKFB3	CAGTCCTGAAACTGACGC	GACAGCCTCTGACCTCTC
GLUT-1	GCCTGAGACCAGTTGAAAGCAC	CTGCTTAGGTAAAGTTACAGGAG
VEGF	TTACTGCTGTACCTCCACC	ACAGGACGGCTTGAAGATG
EPO	AGGGTCACGAAGCCATGAAG	GATTTCGGCTGTTGCCAGTG
HIF-1α	GGTGGATATGTCTGGGTTGAG	TTCAACTGGTTTGAGGACAGA
β-CATENIN	AGGGGTCCTCTGTGAACTTG	CAGCAGTCTCATTCCAAGCC
WNT3a	ATTTGGAGGAATGGTCTCTCG	GCAGGTCTTCACTTCGCAAC
AXIN2	CGCTAGGCGGAATGAAGA	GTATGCACCATCTTGGTC
GAPDH	CAGTGCCAGCCTCGTCTCAT	TGGTAACCAGGCGTCCGATG
β-ACTIN	AGGGAAATCGTGCGTGAC	CGCTCATTGCCGATAGTG
α-TUBULIN	GGTTGAGCCCTACAATTCC	CAATGTCGAGGTTTCTACG
**Site-Directed Mutagenesis**		
**Mutants**	**Forward (5′–3′)**	**Reverse (5′-3′)**
T1492D	TCCAGCACGAAGGCCgacCTGTACCCGCCGATC	GATCGGCGGGTACAGgtcGGCCTTCGTGCTGGA
S1503A	ATCCTGAACCCGCCGCCCgCCCCGGCCACGGACCCCT	AGGGGTCCGTGGCCGGGGcGGGCGGCGGGTTCAGGAT
T1541D	AATGGCGCCCCCGACGgacCCCTGCAGCACCGACG	CGTCGGTGCTGCAGGGgtcCGTCGGGGGCGCCATT
T1578D	TATCCACCCCCACCCgacCCCCACAGCCAGTAC	GTACTGGCTGTGGGGgtcGGGTGGGGGTGGATA
S1598A	GGACAGCTGCCCGCCCgCGCCCGCCACCGAGAG	CTCTCGGTGGCGGGCGcGGGCGGGCAGCTGTCC
S1609A	CTTCCCGCCCCCTCCGgCCCCCTGCACGGACTC	GAGTCCGTGCAGGGGGcCGGAGGGGGCGGGAAG
**Oligos for siRNA**		
**Gene**	**Sequence (5′–3′)**	
PHD1	GCUAGCAUCGGGACAGAAAGG	
PHD2	GCGGAGGUAUUCUUCGAAUUU	
PHD3	GGCUGGAUCUGGAGAAGAUCG	
Negative control	UUCUCCGAACGUGUCACGU	

## Data Availability

The data presented in this study are available on request from the corresponding author. The data are not publicly available due to privacy.

## Supplementary Materials

The following are available online at https://www.mdpi.com/article/10.3390/ijms22126581/s1.

## Abbreviations

Bnip3l	BCL2/adenovirus E1B interacting protein 3-like
BSA	Bovine serum albumin
CAD	C-terminal transactivation domain
CHX	Cycloheximide
Cx43	Connexin 43
DMOG	Dimethyloxalylglycine
EPO	Erythropoietin
FBS	Fetal bovine serum
FIH	Factor inhibiting HIF
Glut-1	Glucose transporter-1
GSK3β	Glycogen synthase kinase 3β
HIF-1α	Hypoxia-inducible factor-1α
HREs	Hypoxia-response elements
ICD	Intracellular domain
I/R	Ischemia/reperfusion
LDLR	Low-density lipoprotein receptor
LRP5	Low-density lipoprotein receptor-related protein 5
MI	Myocardial infarction
PHD2	Prolyl hydroxylase 2
PMSF	Phenylmethylsulfonyl fluoride
TCF	T-cell factor
α-MEM	Alpha-minimum essential medium

## References

[1] Semenza G.L. (1998). Hypoxia-inducible factor 1: Master regulator of O2 homeostasis. Curr. Opin. Genet. Dev..

[2] Harris A.L. (2002). Hypoxia—A key regulatory factor in tumour growth. Nat. Rev. Cancer.

[3] Kumar H., Choi D.K. (2015). Hypoxia Inducible Factor Pathway and Physiological Adaptation: A Cell Survival Pathway?. Mediat. Inflamm..

[4] Semenza G.L. (2001). HIF-1 and mechanisms of hypoxia sensing. Curr. Opin. Cell Biol..

[5] Epstein A.C., Gleadle J.M., McNeill L.A., Hewitson K.S., O’Rourke J., Mole D.R., Mukherji M., Metzen E., Wilson M.I., Dhanda A. (2001). *C. elegans* EGL-9 and mammalian homologs define a family of dioxygenases that regulate HIF by prolyl hydroxylation. Cell.

[6] Maxwell P.H., Wiesener M.S., Chang G.W., Clifford S.C., Vaux E.C., Cockman M.E., Wykoff C.C., Pugh C.W., Maher E.R., Ratcliffe P.J. (1999). The tumour suppressor protein VHL targets hypoxia-inducible factors for oxygen-dependent proteolysis. Nature.

[7] Lando D., Peet D.J., Whelan D.A., Gorman J.J., Whitelaw M.L. (2002). Asparagine hydroxylation of the HIF transactivation domain a hypoxic switch. Science.

[8] Hewitson K.S., McNeill L.A., Riordan M.V., Tian Y.M., Bullock A.N., Welford R.W., Elkins J.M., Oldham N.J., Bhattacharya S., Gleadle J.M. (2002). Hypoxia-inducible factor (HIF) asparagine hydroxylase is identical to factor inhibiting HIF (FIH) and is related to the cupin structural family. J. Biol. Chem..

[9] Mahon P.C., Hirota K., Semenza G.L. (2001). FIH-1: A novel protein that interacts with HIF-1alpha and VHL to mediate repression of HIF-1 transcriptional activity. Genes Dev..

[10] Schofield C.J., Ratcliffe P.J. (2004). Oxygen sensing by HIF hydroxylases. Nat. Rev. Mol. Cell Biol..

[11] Wenger R.H. (2002). Cellular adaptation to hypoxia: O2-sensing protein hydroxylases, hypoxia-inducible transcription factors, and O2-regulated gene expression. FASEB J..

[12] Eckle T., Kohler D., Lehmann R., El Kasmi K., Eltzschig H.K. (2008). Hypoxia-inducible factor-1 is central to cardioprotection: A new paradigm for ischemic preconditioning. Circulation.

[13] Natarajan R., Salloum F.N., Fisher B.J., Kukreja R.C., Fowler A.A. (2006). Hypoxia inducible factor-1 activation by prolyl 4-hydroxylase-2 gene silencing attenuates myocardial ischemia reperfusion injury. Circ. Res..

[14] Wang W.E., Yang D., Li L., Wang W., Peng Y., Chen C., Chen P., Xia X., Wang H., Jiang J. (2013). Prolyl hydroxylase domain protein 2 silencing enhances the survival and paracrine function of transplanted adipose-derived stem cells in infarcted myocardium. Circ. Res..

[15] Pinson K.I., Brennan J., Monkley S., Avery B.J., Skarnes W.C. (2000). An LDL-receptor-related protein mediates Wnt signalling in mice. Nature.

[16] Tamai K., Semenov M., Kato Y., Spokony R., Liu C., Katsuyama Y., Hess F., Saint-Jeannet J.P., He X. (2000). LDL-receptor-related proteins in Wnt signal transduction. Nature.

[17] Laine C.M., Chung B.D., Susic M., Prescott T., Semler O., Fiskerstrand T., D’Eufemia P., Castori M., Pekkinen M., Sochett E. (2011). Novel mutations affecting LRP5 splicing in patients with osteoporosis-pseudoglioma syndrome (OPPG). Eur. J. Hum. Genet..

[18] Bjorklund P., Svedlund J., Olsson A.K., Akerstrom G., Westin G. (2009). The internally truncated LRP5 receptor presents a therapeutic target in breast cancer. PLoS ONE.

[19] Li Y., Lu W., He X., Schwartz A.L., Bu G. (2004). LRP6 expression promotes cancer cell proliferation and tumorigenesis by altering beta-catenin subcellular distribution. Oncogene.

[20] Borrell-Pages M., Romero J.C., Badimon L. (2015). LRP5 deficiency down-regulates Wnt signalling and promotes aortic lipid infiltration in hypercholesterolaemic mice. J. Cell. Mol. Med..

[21] Alfaro M.P., Pagni M., Vincent A., Atkinson J., Hill M.F., Cates J., Davidson J.M., Rottman J., Lee E., Young P.P. (2008). The Wnt modulator sFRP2 enhances mesenchymal stem cell engraftment, granulation tissue formation and myocardial repair. Proc. Natl. Acad. Sci. USA.

[22] Barandon L., Casassus F., Leroux L., Moreau C., Allieres C., Lamaziere J.M., Dufourcq P., Couffinhal T., Duplaa C. (2011). Secreted frizzled-related protein-1 improves postinfarction scar formation through a modulation of inflammatory response. Arterioscler. Thromb. Vasc. Biol..

[23] Zhang Z., Deb A., Zhang Z., Pachori A., He W., Guo J., Pratt R., Dzau V.J. (2009). Secreted frizzled related protein 2 protects cells from apoptosis by blocking the effect of canonical Wnt3a. J. Mol. Cell. Cardiol..

[24] Saraswati S., Alfaro M.P., Thorne C.A., Atkinson J., Lee E., Young P.P. (2010). Pyrvinium, a potent small molecule Wnt inhibitor, promotes wound repair and post-MI cardiac remodeling. PLoS ONE.

[25] Wo D., Peng J., Ren D.N., Qiu L., Chen J., Zhu Y., Yan Y., Yan H., Wu J., Ma E. (2016). Opposing Roles of Wnt Inhibitors IGFBP-4 and Dkk1 in Cardiac Ischemia by Differential Targeting of LRP5/6 and beta-catenin. Circulation.

[26] Abe T., Zhou P., Jackman K., Capone C., Casolla B., Hochrainer K., Kahles T., Ross M.E., Anrather J., Iadecola C. (2013). Lipoprotein receptor-related protein-6 protects the brain from ischemic injury. Stroke.

[27] Borrell-Pages M., Vilahur G., Romero J.C., Casani L., Bejar M.T., Badimon L. (2016). LRP5/canonical Wnt signalling and healing of ischemic myocardium. Basic Res. Cardiol..

[28] MacDonald B.T., Semenov M.V., Huang H., He X. (2011). Dissecting molecular differences between Wnt coreceptors LRP5 and LRP6. PLoS ONE.

[29] MacDonald B.T., Tamai K., He X. (2009). Wnt/beta-catenin signaling: Components, mechanisms, and diseases. Dev. Cell.

[30] Chin E.N., Martin J.A., Kim S., Fakhraldeen S.A., Alexander C.M. (2015). Lrp5 Has a Wnt-Independent Role in Glucose Uptake and Growth for Mammary Epithelial Cells. Mol. Cell. Biol..

[31] Li J., Li C., Liang D., Lv F., Yuan T., The E., Ma X., Wu Y., Zhen L., Xie D. (2016). LRP6 acts as a scaffold protein in cardiac gap junction assembly. Nat. Commun..

[32] Fujino T., Asaba H., Kang M.J., Ikeda Y., Sone H., Takada S., Kim D.H., Ioka R.X., Ono M., Tomoyori H. (2003). Low-density lipoprotein receptor-related protein 5 (LRP5) is essential for normal cholesterol metabolism and glucose-induced insulin secretion. Proc. Natl. Acad. Sci. USA.

[33] Clevers H., Nusse R. (2012). Wnt/beta-catenin signaling and disease. Cell.

[34] Freeman R.S., Hasbani D.M., Lipscomb E.A., Straub J.A., Xie L. (2003). SM-20, EGL-9, and the EGLN family of hypoxia-inducible factor prolyl hydroxylases. Mol. Cells.

[35] Villar D., Vara-Vega A., Landazuri M.O., Del Peso L. (2007). Identification of a region on hypoxia-inducible-factor prolyl 4-hydroxylases that determines their specificity for the oxygen degradation domains. Biochem. J..

[36] Berra E., Benizri E., Ginouves A., Volmat V., Roux D., Pouyssegur J. (2003). HIF prolyl-hydroxylase 2 is the key oxygen sensor setting low steady-state levels of HIF-1alpha in normoxia. EMBO J..

[37] Daskalopoulos E.P., Hermans K.C., Janssen B.J., Blankesteijn W.M. (2013). Targeting the Wnt/frizzled signaling pathway after myocardial infarction: A new tool in the therapeutic toolbox?. Trends Cardiovasc. Med..

[38] Aisagbonhi O., Rai M., Ryzhov S., Atria N., Feoktistov I., Hatzopoulos A.K. (2011). Experimental myocardial infarction triggers canonical Wnt signaling and endothelial-to-mesenchymal transition. Dis. Models Mech..

[39] Paik D.T., Rai M., Ryzhov S., Sanders L.N., Aisagbonhi O., Funke M.J., Feoktistov I., Hatzopoulos A.K. (2015). Wnt10b Gain-of-Function Improves Cardiac Repair by Arteriole Formation and Attenuation of Fibrosis. Circ. Res..

[40] Zelarayan L.C., Noack C., Sekkali B., Kmecova J., Gehrke C., Renger A., Zafiriou M.P., van der Nagel R., Dietz R., de Windt L.J. (2008). Beta-Catenin downregulation attenuates ischemic cardiac remodeling through enhanced resident precursor cell differentiation. Proc. Natl. Acad. Sci. USA.

[41] van de Schans V.A., van den Borne S.W., Strzelecka A.E., Janssen B.J., van der Velden J.L., Langen R.C., Wynshaw-Boris A., Smits J.F., Blankesteijn W.M. (2007). Interruption of Wnt signaling attenuates the onset of pressure overload-induced cardiac hypertrophy. Hypertension.

[42] Woulfe K.C., Gao E., Lal H., Harris D., Fan Q., Vagnozzi R., DeCaul M., Shang X., Patel S., Woodgett J.R. (2010). Glycogen synthase kinase-3beta regulates post-myocardial infarction remodeling and stress-induced cardiomyocyte proliferation in vivo. Circ. Res..

[43] Min J.K., Park H., Choi H.J., Kim Y., Pyun B.J., Agrawal V., Song B.W., Jeon J., Maeng Y.S., Rho S.S. (2011). The WNT antagonist Dickkopf2 promotes angiogenesis in rodent and human endothelial cells. J. Clin. Investig..

[44] Zhong Z., Baker J.J., Zylstra-Diegel C.R., Williams B.O. (2012). Lrp5 and Lrp6 play compensatory roles in mouse intestinal development. J. Cell. Biochem..

[45] Joeng K.S., Schumacher C.A., Zylstra-Diegel C.R., Long F., Williams B.O. (2011). Lrp5 and Lrp6 redundantly control skeletal development in the mouse embryo. Dev. Biol..

[46] Mi K., Johnson G.V. (2005). Role of the intracellular domains of LRP5 and LRP6 in activating the Wnt canonical pathway. J. Cell. Biochem..

[47] Holmen S.L., Giambernardi T.A., Zylstra C.R., Buckner-Berghuis B.D., Resau J.H., Hess J.F., Glatt V., Bouxsein M.L., Ai M., Warman M.L. (2004). Decreased BMD and limb deformities in mice carrying mutations in both Lrp5 and Lrp6. J. Am. Soc. Bone Miner. Res..

[48] Joiner D.M., Ke J., Zhong Z., Xu H.E., Williams B.O. (2013). LRP5 and LRP6 in development and disease. Trends Endocrinol. Metab. TEM.

[49] Kelly O.G., Pinson K.I., Skarnes W.C. (2004). The Wnt co-receptors Lrp5 and Lrp6 are essential for gastrulation in mice. Development.

[50] Appelhoff R.J., Tian Y.M., Raval R.R., Turley H., Harris A.L., Pugh C.W., Ratcliffe P.J., Gleadle J.M. (2004). Differential function of the prolyl hydroxylases PHD1, PHD2, and PHD3 in the regulation of hypoxia-inducible factor. J. Biol. Chem..

[51] Serocki M., Bartoszewska S., Janaszak-Jasiecka A., Ochocka R.J., Collawn J.F., Bartoszewski R. (2018). miRNAs regulate the HIF switch during hypoxia: A novel therapeutic target. Angiogenesis.

[52] Holmquist L., Jögi A., Påhlman S. (2005). Phenotypic persistence after reoxygenation of hypoxic neuroblastoma cells. Int. J. Cancer.

[53] Holmquist-Mengelbier L., Fredlund E., Löfstedt T., Noguera R., Navarro S., Nilsson H., Pietras A., Vallon-Christersson J., Borg A., Gradin K. (2006). Recruitment of HIF-1alpha and HIF-2alpha to common target genes is differentially regulated in neuroblastoma: HIF-2alpha promotes an aggressive phenotype. Cancer Cell.

[54] Bartoszewski R., Moszyńska A., Serocki M., Cabaj A., Polten A., Ochocka R., Dell’Italia L., Bartoszewska S., Króliczewski J., Dąbrowski M. (2019). Primary endothelial cell-specific regulation of hypoxia-inducible factor (HIF)-1 and HIF-2 and their target gene expression profiles during hypoxia. FASEB J..

[55] Koh M.Y., Lemos R., Liu X., Powis G. (2011). The hypoxia-associated factor switches cells from HIF-1α- to HIF-2α-dependent signaling promoting stem cell characteristics, aggressive tumor growth and invasion. Cancer Res..

[56] Lin Q., Cong X., Yun Z. (2011). Differential hypoxic regulation of hypoxia-inducible factors 1alpha and 2alpha. Mol. Cancer Res..

[57] Uchida T., Rossignol F., Matthay M.A., Mounier R., Couette S., Clottes E., Clerici C. (2004). Prolonged hypoxia differentially regulates hypoxia-inducible factor (HIF)-1alpha and HIF-2alpha expression in lung epithelial cells: Implication of natural antisense HIF-1alpha. J. Biol. Chem..

[58] Oerlemans M.I., Goumans M.J., van Middelaar B., Clevers H., Doevendans P.A., Sluijter J.P. (2010). Active Wnt signaling in response to cardiac injury. Basic Res. Cardiol..

[59] Staal F.J., Luis T.C., Tiemessen M.M. (2008). WNT signalling in the immune system: WNT is spreading its wings. Nat. Rev. Immunol..

[60] Holdstock L., Meadowcroft A.M., Maier R., Johnson B.M., Jones D., Rastogi A., Zeig S., Lepore J.J., Cobitz A.R. (2016). Four-Week Studies of Oral Hypoxia-Inducible Factor-Prolyl Hydroxylase Inhibitor GSK1278863 for Treatment of Anemia. J. Am. Soc. Nephrol..

[61] Ju S., Park S., Lim L., Choi D.H., Song H. (2020). Low density lipoprotein receptor-related protein 1 regulates cardiac hypertrophy induced by pressure overload. Int. J. Cardiol..

[62] Chang W., Song B.W., Lim S., Song H., Shim C.Y., Cha M.J., Ahn D.H., Jung Y.G., Lee D.H., Chung J.H. (2009). Mesenchymal stem cells pretreated with delivered Hph-1-Hsp70 protein are protected from hypoxia-mediated cell death and rescue heart functions from myocardial injury. Stem Cells.

[63] Song H., Song B.W., Cha M.J., Choi I.G., Hwang K.C. (2010). Modification of mesenchymal stem cells for cardiac regeneration. Expert Opin. Biol. Ther..

[64] Pfaffl M.W. (2001). A new mathematical model for relative quantification in real-time RT-PCR. Nucleic Acids Res..

[65] Song H., Hwang H.J., Chang W., Song B.W., Cha M.J., Kim I.K., Lim S., Choi E.J., Ham O., Lee C.Y. (2011). Cardiomyocytes from phorbol myristate acetate-activated mesenchymal stem cells restore electromechanical function in infarcted rat hearts. Proc. Natl. Acad. Sci. USA.

