# Supplementary material for: LRP5 Regulates HIF-1α Stability via Interaction with PHD2 in Ischemic Myocardium
Source: Int J Mol Sci. 2021 Jun 19;22(12):6581. doi: 10.3390/ijms22126581 (PMC8235097; doi:10.3390/ijms22126581)
Supplement: Supplementary file 1 [file ijms-22-06581-s001.zip › ijms-1249051-supplementary.pdf]

Supplementary Data

Figure S1. Altered expression of LRP5 in cardiomyocytes under hypoxia

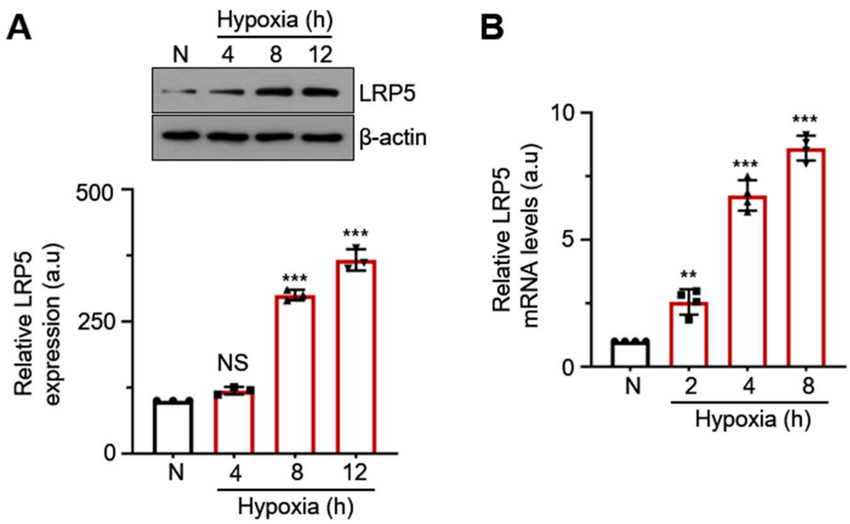

Figure S1. Cardiomyocytes were exposed to normoxia (indicated by "N") or hypoxia (indicated by "H") (O_2 1.2%) at the indicated times. (**A**) The expression levels of LRP5 were analyzed on western blotting. Protein levels were normalized to β-actin levels. n=3/group. (**B**) The mRNA levels of LRP5 were quantified using qPCR. Gene expression was normalized to GAPDH. n=4/group. All graphs show means ± SD. **P < 0.01, ***P < 0.001 versus Normoxia; NS, no significance.

Figure S2. Construction of LRP5/6 and shLRP5/6 adenoviral vectors and their effects on the viability of hypoxic cardiomyocytes

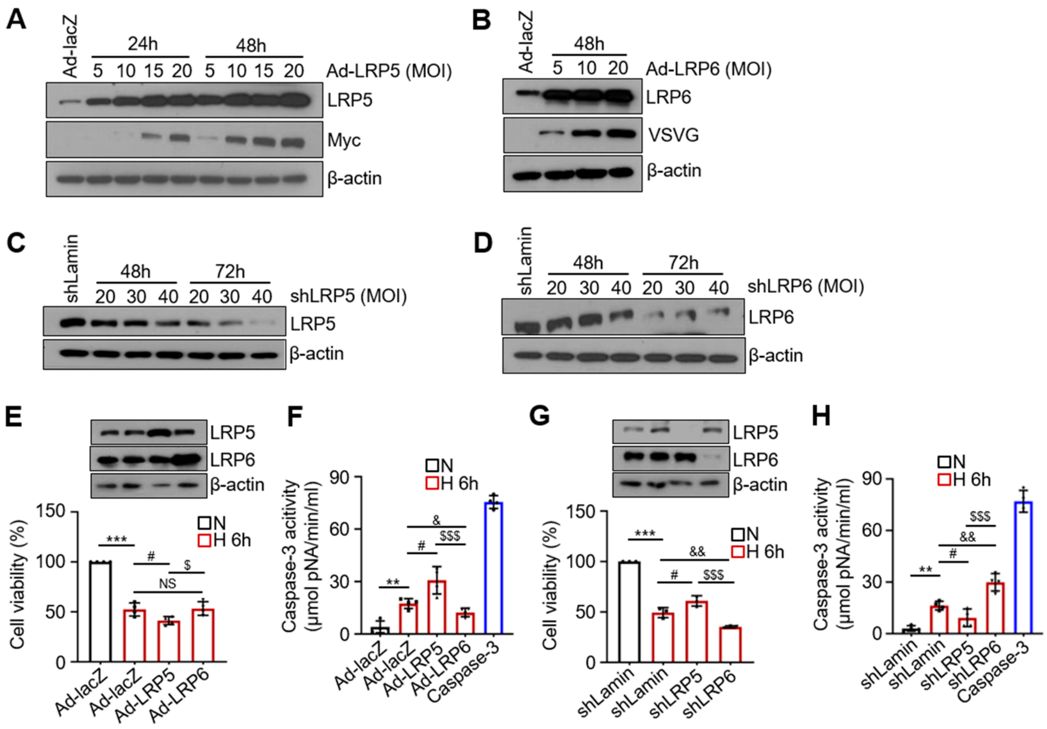

Figure S2. Cardiomyocytes were infected with adenoviruses expressing full-length LRP5/6 or shRNA targeting LRP5/6 at the indicated MOI values and times. Adenoviral transduction efficiency was assessed using western blotting. (**A**) Representative western blot images of cells infected with Ad-LRP5 (Myc tagging). (**B**) Representative western blot images of cells infected with Ad-LRP6 (VSVG tagging). (**C**) Representative western blot images of cells infected with shLRP5. (**D**) Representative western blot images of cells infected with shLRP6. Representative immunoblots from two independent experiments are presented. (**E**) Relative percent of cell viability was assessed using MTT assay in Ad-lacZ-, Ad-LRP5-, and Ad-LRP6-infected hypoxic cardiomyocytes. Normoxia is indicated as "N" and hypoxia is indicated as "H". Cell viability was normalized to normoxic Ad-lacZ control. n=4/group. Means ± SD. $^{***}P < 0.001$ versus Normoxia; $^{\#}P < 0.05$ Ad-LRP5 versus Ad-lacZ; $^{\$}P < 0.05$ Ad-LRP6 versus Ad-LRP5; NS, no significance. (**F**) Measurement of caspase-3 activity for Ad-lacZ-, Ad-LRP5-, and Ad-LRP6-infected hypoxic cardiomyocytes. Data are presented in µmol of pNA (p-Nitroaniline) per minute per volume of sample in ml. Caspase-3 was used as a positive control; 5 µl of 5 µg/ml. n=4/group. Means ± SD. $^{**}P < 0.01$ versus Normoxia; $^{\#}P < 0.05$ Ad-LRP5 versus Ad-lacZ; $^{\$\$\$}P < 0.001$ Ad-LRP6 versus Ad-LRP5; $^{\&}P < 0.05$ Ad-LRP6 versus Ad-lacZ. (**G**) Relative percent of cell viability assessed using MTT assay in shLamin-, shLRP5-, and shLRP6-infected hypoxic cardiomyocytes. Cell viability was normalized to normoxic shLamin control. G; n=4; H; n=3. Means ± SD. $^{**}P < 0.01$ versus Normoxia; $^{\#}P < 0.05$ shLRP5 versus shLamin; $^{\$\$\$}P < 0.001$ shLRP6 versus shLRP5; $^{\&}P < 0.05$ shLRP6 versus shLamin. (**H**) Measurement of caspase-3 activity for shLamin-, shLRP5-, and shLRP6-infected hypoxic cardiomyocytes. n=4/group. Means ± SD. $^{**}P < 0.01$ versus Normoxia; $^{\#}P < 0.05$ shLRP5 versus shLamin; $^{\$\$\$}P < 0.001$ shLRP6 versus shLRP5; $^{\&\&}P < 0.01$ shLRP6 versus shLamin.

Figure S3. LRP5 does not regulate HIF-1α expression at the transcriptional level but inhibits nuclear translocation of HIF-1α

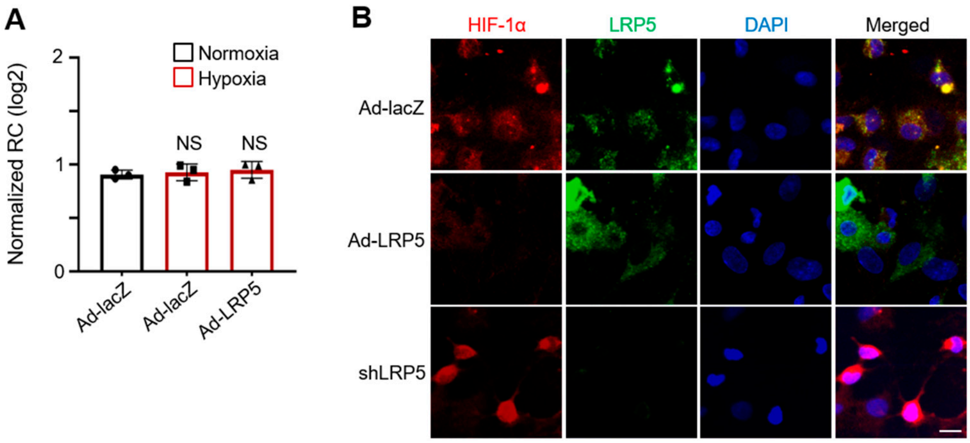

Figure S3. (**A**) Extracted total RNA from triplicated biological samples were subjected to mRNA sequencing. Each Fold change in hypoxic Ad-lacZ and Ad-LRP5-infected cardiomyocytes was calculated relative to normoxic Ad-lacZ control. n=3/group. Graph shows means ± SD. NS, no significance. (**B**) Ad-lacZ-, Ad-LRP5-, and shLRP5-infected cardiomyocytes were co-stained with HIF-1α (red) and LRP5 (green) after exposure to hypoxic condition (90 min). Nuclei were stained with DAPI (blue). The image shows translocation of HIF-1α into the nucleus. Immunofluorescence was observed using confocal microscopy. Scale bar, 50 μm.

Figure S4. Altered expression of Wnt3a in LRP5-silenced cardiomyocytes and effects of hypoxia on β-catenin expression

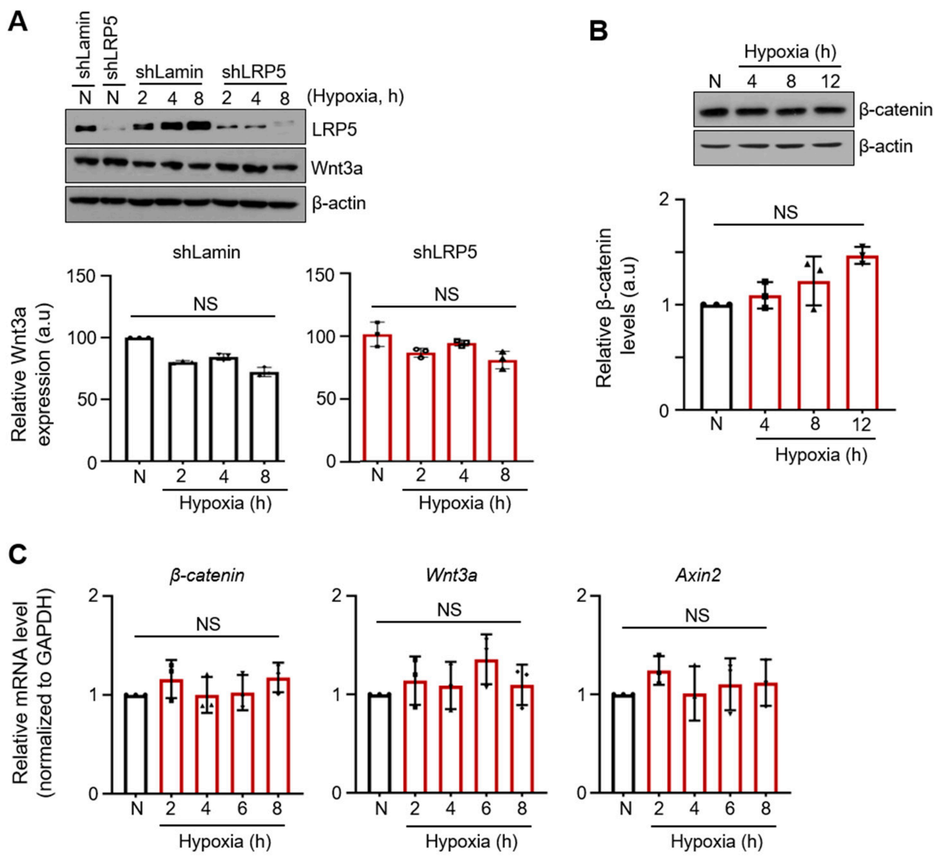

Figure S4. (**A**) Cardiomyocytes were exposed to normoxia or hypoxia (O_2 3%) at the indicated times. Then protein expression levels were analyzed on western blotting and their levels were normalized to β-actin levels. Protein levels were normalized to β-actin levels. n=3/group. All graphs show means ± SD. NS, no significance. (**B** and **C**) Cardiomyocytes were exposed to hypoxia and harvested at the indicated time points for (**B**) western blotting and (**C**) real-time qPCR. Protein and gene expression was normalized to β-actin and GAPDH, respectively. n=3/group. All graphs show means ± SD. NS, no significance.

Figure S5. *In vivo* **myocardial gene delivery using adenoviruses**

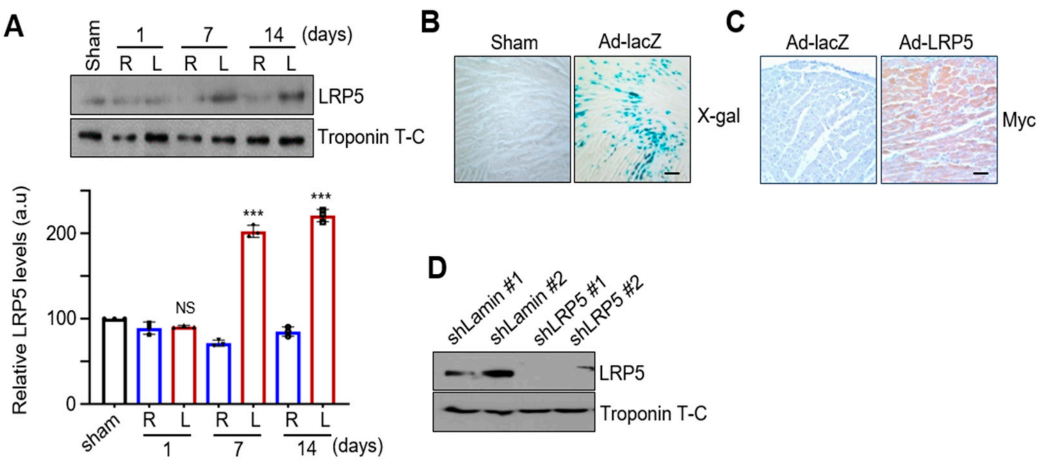

Figure S5. (**A**) Representative western blot analysis showing expression of LRP5 form right and left ventricle in I/R-injured heart lysates. Troponin T-C was used as the loading control. n=3/group. Graph shows means ± SD. ***P < 0.001 versus right venticle; NS, no significance. (**B**) Representative sections from Sham or lacZ-infected heart tissues stained with X-gal (blue) to assess β-galactosidase activity. n=3/group. Scale bar, 100 µm. (**C**) Representative images of immunohistochemical staining from lacZ or LRP5-infected heart tissues using Myc antibody. n=3/group. Scale bar, 100 µm. (**D**) Representative western blot analysis showing expression of LRP5 in shLamin- or shLRP5-injected heart lysates. Troponin T-C was used as the loading control.

Table S1. Functional enrichment analysis of common genes by GO-terms.

A. Ad-lacZ

Term	Category	Number of genes	%	p-value	Genes
Cellular response to hypoxia	B.P.	25	2.3	1.8E-9	Bnip3, Bnip3l, Bbc3, Fas, Higd1a, Mdm2, Ndrg1, Ppargc1a, Rora, Cox4i2, Ero1a, Fam162a, Foxo3, Hmox1, IL7, Mif, Mgarp, Kcnk3, Slc2a4, Slc8a3, Stc1, Stc2, Tbl2, Vegfa, Vldlr
Response to hypoxia	B.P.	35	3.3	6.9E-9	Abcb1b, Bnip3, Cxcr4, Cx3cl1, Cbfa2t3, Cd24, Ddit4, Fosl2, Fas, Ppargc1a, Xrcc1, Aldh3a1, Aldoc, Alkbh5, Ang, Angpt2, Angptl4, Apold1, Cdkn1b, Cyp1a1, Egln1, Egln3, Ednra, Hp, Hmox1, Il1a, Il1b, Kdm3a, Nos2, Nfatc3, Ppara, Pkm, Tgfbr3, Vegfa, Vldlr
Glycolytic process	B.P.	10	0.9	2.3E-5	Aldoc, Eno2, LOC100911625, Gpi, Hk2, Pfkl, Pkm, Tpi1, LOC500959, LOC100911515
Negative regulation of apoptotic process	B.P.	37	3.5	1.5E-3	Alms1, Ahi1, Bcl6, Bnip3, Bnip3l, Fas, Gli2, Higd1a, Mdm2, Mgmt, Pim1, Wt1, Amigo2, Angptl4, Alox15, Birc3, Cdkn1a, Cdkn1b, Dapk1, Ednra, Eef2k, Eif2ak2, Gnrh1, Gfer, Ifit3, Il6, Il7, Kdm2a, Ltk, Mif, Slc2a3, LOC100909595, Slc25a27, Slc40a1, Sycp2, Sycp3, Vegfa
Positive regulation of apoptotic process	B.P.	26	2.4	3.5E-3	Bcl6, Bcl2l11, Bnip3, Bnip3l, Bbc3, Bmf, Ddit3, Fas, Wt1, Aldh1a3, Dhodh, Egln1, Eif2ak2, Fam162a, Foxo3, Hmox1, Il1b, Il6, Jmy, Map3k1, Nos2, Nfatc4, Osgin1, Scin, Tfap4, Tp53inp1
Oxidation-reduction process	B.P.	43	4.0	2.8E-3	Foxred2, Hhipl1, Higd1a, Ndufa4l2, Aldh1a3, Aldh1l1, Aldh3a1, Aox1, Aox2, Akr1c19, Bco1, Cbr1, Cbr3, LOC102556347, Cyp1a1, Cyp2r1, Cyp27a1, Cyp39a1, Cyp4f37, Cyp4f39, Cyp4f5, Cyp7b1, Cyb5r2, Dhrs3, Dhdh, Dhodh, Egln1, Egln3, Ero1a, Gsto2, Gfer, Hsd17b13, Kdm3a, Kdm5b, Maob, Nos2, Osgin1, P4ha2, Spr, LOC497963, RGD1560795, Smox, Srxn1
Negative regulation of transcription from RNA polymerase II promoter	B.P.	47	4.4	4.3E-3	Bcl6, Cbfa2t3, Crebrf, Ddit3, Gzf1, Gli2, Glis1, Glis3, Mxi1, Mnt, Mdm2, Phf21a, Rcor2, LOC102552640, Sox6, Sap30, Wt1, Atn1, Bhlhe40, Cbx2, Cbx7, Cdkn1c, Cpeb3, Ezh1, Efna1, Fgf9, Fnip1, Foxo3, Hist1h1c, Hdac9, Il1b, Lcor, Lpin1, Mxd1, Nfatc4, Ncoa2, Nr1d1, Ppara, Sik1, Sqstm1, Stat1, Sorbs3, Tshz2, Vegfa, Vldlr, LOC102550291, Zhx2
Apoptotic process	B.P.	23	2.1	5.0E-2	Bnip3, Bbc3, Ddit3, Ddit4, Fas, Pim1, Tnfrsf9, Wt1, XAF1, Aldoc, Birc3, Dapk1, Egln3, Foxo3, Gas2, Gja1, Irf1, Mapt, Niacr1, Rnf152, Sqstm1, Tp53inp1, Usp53
Glucose homeostasis	B.P.	12	1.1	1.3E-2	Alms1, Csmd1, Adgrf5, Foxo3, Gpi, Insr, Il6, Pde3b, Pygl, Ppp1r3g, Pdk4, Slc2a4

B. Ad-LRP5

Term	Category	Number of genes	%	p-value	Genes
Cellular response to hypoxia	B.P.	20	2.3	7.9E-8	Bnip3, Bnip3l, Bbc3, Fas, Higd1a, Mdm2, Ndrg1, Rora, Cox4i2, Ero1a, Fam162a, Foxo3, Hmox1, IL7, Mif, Mgarp, Slc8a3, Stc2, Tbl2, Vegfa
Response to hypoxia	B.P.	28	3.3	1.8E-7	Abcb1b, Bnip3, Cxcr4, Cx3cl1, Cbfa2t3, Cd24, Ddit4, Fosl2, Fas, Aldh3a1, Aldoc, Alkbh5, Angpt2, Angptl4, Apold1, Cdkn1b, Cyp1a1, Egln1, Egln3, Ednra, Hp, Hmox1, Il1a, Il1b, Kdm3a, Nos2, Tgfbr3, Vegfa
Glycolytic process	B.P.	6	0.7	7.8E-3	Aldoc, Eno2, LOC100911625, Gpi, Hk2, Pfkl, Pkm, Tpi1, LOC500959, LOC100911515
Negative regulation of apoptotic process	B.P.	32	3.7	6.5E-4	Ahi1, Bcl6, Bnip3, Bnip3l, Fas, Gli2, Higd1a, Mdm2, Mgmt, Pim1, Wt1, Amigo2, Angptl4, Alox15, Birc3, Cdkn1b, Dapk1, Ednra, Eif2ak2, Gnrh1, Ifit3, Il6, Il7, Ltk, Mif, Pde3a, Slc2a3, LOC100909595, Slc25a27, Sycp2, Sycp3, Vegfa
Positive regulation of apoptotic process	B.P.	27	3.1	3.7E-5	Bcl6, Bcl2l11, Bnip3, Bnip3l, Bbc3, Bmf, Ddit3, Fas, Wt1, Aldh1a3, Dhodh, Egln1, Eif2ak2, Fam162a, Foxo3, Hmox1, Il1b, Il6, Jmy, Map3k1, Ntrk3, Ntf3, Nos2, Nfatc4, Scin, Tfap4, Tp53inp1
Oxidation-reduction process	B.P.	36	4.2	2.0E-3	Foxred2, Hhipl1, Higd1a, Ndufa4l2, Aldh1a3, Aldh1l1, Aldh3a1, Aox1, Aox2, Akr1c19, Bco1, Cbr1, Cbr3, LOC102556347, Cyp1a1, Cyp2r1, Cyp27a1, Cyp39a1, Cyp4f37, Cyp4f39, Cyp4f5, Cyp7b1, Cyb5r2, Dhrs3, Dhdh, Dhodh, Egln1, Egln3, Ero1a, Gsto2, Gfer, Hsd17b13, Kdm3a, Kdm5b, Maob, Nos2, Osgin1, P4ha2, Spr, LOC497963, RGD1560795, Smox, Srxn1
Negative regulation of transcription from RNA polymerase II promoter	B.P.	37	4.3	9.6E-3	Bcl6, Cbfa2t3, Crebrf, Ddit3, Gli2, Glis1, Glis3, Mxi1, Mnt, Mdm2, Phf21a, Rcor2, LOC102552640, Wt1, Atn1, Bhlhe40, Cbx2, Cbx7, Ezh1, Efna1, Fgf9, Fnip1, Foxo3, Il1b, Lcor, Lpin1, Mxd1, Nfatc4, Ncoa2, Sik1, Sqstm1, Stat1, Sorbs3, Tshz2, Vegfa, LOC102550291, Zhx2
Apoptotic process	B.P.	20	2.3	2.7E-2	Bnip3, Bbc3, Ddit3, Ddit4, Fas, Pim1, Tnfrsf9, Wt1, XAF1, Aldoc, Birc3, Dapk1, Egln3, Foxo3, Gas2, Irf1, Niacr1, Rnf152, Sqstm1, Tp53inp1
Glucose homeostasis	B.P.	9	1.0	4.4E-2	Adgrf5, Foxo3, Gpi, Insr, Il6, Pde3b, Pygl, Ppp1r3g, Pdk4

C. shLRP5

Term	Category	Number of genes	%	p-value	Genes
Response to hypoxia	B.P.	40	3.5	7.0E-11	Abcb1b, Bnip3, Cxcr4, Cx3cl1, Cbfa2t3, Cd24, Ddit4, Fosl2, Fas, Ppargc1a, Xrcc1, Aldh3a1, Aldoc, Alkbh5, Ang, Angpt2, Angptl4, Apold1, Car9, Cdkn1b, Cyp1a1, Egln1, Egln3, Ednra, Hp, Hmox1, Kdm3a, Nos1, Nos2, Nfatc3, Ppara, Plod1, Penk, P2rx3, Pkm, Sod3, Trh, Tgfbr3, Vegfa, Vldlr
Cellular response to hypoxia	B.P.	26	2.3	1.5E-9	Atp7a, Bnip3, Bnip3l, Bbc3, Fas, Higd1a, Mdm2, Ndrg1, Ppargc1a, Rora, S100b, Cox4i2, Ero1a, Fam162a, Foxo3, Hmox1, Mif, LOC103694877, Mgarp, Slc2a4, Slc8a3, Stc1, Stc2, Tbl2, Vegfa, Vldlr
Oxidation-reduction process	B.P.	52	4.6	5.8E-5	Foxred2, Hhipl1, Higd1a, Ndufa4l2, Aldh1a3, Aldh1l1, Aldh3a1, Aox1, Aox2, Akr1c19, Bco1, Cbr1, Cbr3, LOC102556347, Cyp1a1, Cyp2r1, Cyp27a1, Cyp39a1, Cyp4f37, Cyp4f39, Cyp4f5, Cyp7b1, Cyb5r2, Dhrs3, Dhdh, Dhodh, Egln1, Egln3, Ero1a, Gsto2, Gfer, Hsd17b13, Kdm3a, Kdm5b, Maob, Nos2, Osgin1, P4ha2, Spr, LOC497963, RGD1560795, Smox, Srxn1
Glycolytic process	B.P.	10	0.9	4.0E-5	Aldoc, Eno2, LOC100911625, Gpi, Hk2, Pfkl, Pkm, Tpi1, LOC500959, LOC100911515
Negative regulation of transcription from RNA polymerase II promoter	B.P.	54	4.8	4.8E-4	Bcl6, Cbfa2t3, Crebrf, Ddit3, Gatad2b, Gzf1, Gli2, Glis1, Glis3, Mxi1, Mnt, Mdm2, Phf21a, Rcor2, LOC102552640, Sox18, Sox6, Wt1, Atn1, Acvr2b, Atn1, Bhlhe40, Cbx2, Cbx7, Cdkn1c, Ednrb, Ezh1, Efna1, Fgf9, Fnip1, Foxo3, Foxp2, Hes3, LOC100912068, Hic1, Jarid2, Lcor, Lpin1, Mxd1, Msc, Mzf1, Notch1, Nfatc4, Nr0b2, Nr1d1, Ppara, Kcnip3, Rara, Sik1, Sqstm1, Sorbs3, Tshz2, Vegfa, Vldlr
Positive regulation of apoptotic process	B.P.	31	2.7	2.5E-4	Bcl6, Bcl2l11, Bnip3, Bnip3l, Bbc3, Bmf, Ddit3, Fas, S100b, Steap3, Wt1, C6, Cyp1b1, Dhodh, Egln1, Fam162a, Foxo1, Foxo3, Hmox1, Jmy, Map3k1, Map3k9, Nos2, Notch1, Nfatc4, Osgin1, Scin, Tfap4, Tp53inp1, Uaca, Zfp819
Glucose homeostasis	B.P.	15	1.3	1.1E-3	Csmd1, Adgrf5, Ankrd26, Foxo1, Foxo3, Gcgr, Gpi, Insr, Igfbp5, Pde3b, Pygl, Ppp1r3g, Pdk4, Slc2a4, Slc37a4
Negative regulation of apoptotic process	B.P.	34	3.0	2.3E-2	Ahi1, Bcl6, Bnip3, Bnip3l, Ercc5, Fas, Gli2, Higd1a, Mdm2, Wt1, Amigo2, Angptl4, Alox15, Birc3, Cdkn1b, Dapk1, Dpep1, Ednra, Ednrb, Eef2k, Foxo1, Gnrh1, Ltk, Kdm2a, Mif, Nos1, Pde3a, Pik3r1, Rara, Slc2a3, LOC100909595, Slc40a1, Sycp3, Vegfa
Apoptotic process	B.P.	24	2.1	2.9E-2	Bnip3, Bbc3, Ddit3, Ddit4, Fas, Madd, Steap3, Tnfrsf9, Wt1, Aldoc, Birc3, Chi3l1, Dapk1, Dpep1, Duxbl1, Egln3, Foxo1, Foxo3, Gja1, Kcnip3, Rtkn, Rnf153, Sqstm1, Tp53inp1

GO, gene ontology; BP, biological process.

Table S2. KEGG enrichment analysis of common genes in hypoxic cardiomyocytes.

A. Ad-lacZ

Term	Number of genes	%	p-value	Genes
Metabolic pathways	69	6.4	8.0E-3	Gbe1, Hmgcs2, Nt5c2, Gch1, Nadk2, Ndufa4l2, Rev3l, Ugp2, Ak4, Aldh1a3, Aldh3a1, Aox1, Aox2, Aldoc, Acer2, Aadat, Alox15, Bco1, Cbr1, Cbr3, LOC102556347, Cers6, Cmpk2, Cyp1a1, Cyp2r1, Cyp27a1, Cox4i2, Dhrs3, Dgki, Dhodh, Eno2, LOC100911625, Gpi, Gls2, Gfpt2, Gpat3, Hpse, Hk2, Ipmk, Kyat1, Kyat3, Lpin1, Mboat2, Maob, Mtmr14, Mtmr7, Nampt, Nos2, Olah, Pik3c2b, Pik3c2g, Phospho1, Pfkl, Pla2g2a, Pla2g4b, Pla2g6, Pla2g4f, Pla2g12a, Pygl, P4ha2, Ptges, Pdxp, Pkm, Spr, LOC497963, Tpi1, LOC500959, LOC100911515, Upp1
HIF-1 signaling pathway	40	3.6	2.5E-6	Pfkfb3, Angpt2, Cdkn1a, Cdkn1b, Egln1, Egln3, Eno2, LOC100911625, Hmox1, Hk2, Insr, Nos2, Pdk1, LOC497963, Slc2a1, Vegfa, Bnip3, Ndufa4l2, Cmpk2, Ndrg1, Ak4, Ddit4, Ero1a, Ccng2, Maob, Angptl4, Cox4i2, Cbfa2t3, Olah, Higd1a, Fam162a, Foxo3, pdk1, Nampt, HP, Ang, Ppara, Tpi1, kyat1, Slc2a4
PI3K-Akt signaling pathway	25	2.3	4.7E-3	Bcl2l11, Ddit4, Gng4, Mdm2, Angpt2, Col6a5, Col6a6, Col11a2, Col24a1, Csf3, Cdkn1a, Cdkn1b, Efna1, Fgf11, Fgf9, Foxo3, Gys1, Insr, Il2ra, Il6, Il7, Lama5, Pdgfra, Reln, Vegfa
Cytokine-cytokine receptor interaction	21	2.0	1.3E-4	Ccl5, Cxcl10, Cxcl11, Cxcl13, Cxcl2, Cxcr4, Cx3cl1, Fas, Tnfrsf14, Tnfrsf1b, Tnfrsf9, Ackr3, Csf3, Eda2r, Il1a, Il1b, Il15ra, Il2ra, Il6, Il7, Lif
FoxO signaling pathway	14	1.3	3.1E-3	Bcl6, Bcl2l11, Bnip3, Fbxo32, Gabarapl1, Mdm2, Ccng2, Cdkn1a, Cdkn1b, Foxo3, Insr, Il6, Prkab2, Slc2a4
TNF signaling pathway	15	1.4	1.1E-4	Ccl5, Cxcl1, Cxcl10, Cxcl2, Cx3cl1, Fas, Nfkbia, Tnfaip3, Tnfrsf1b, Traf1, Birc3, Cxcl3, Il1b, Il6, Lif

B. Ad-LRP5

Term	Number of genes	%	p-value	Genes
Metabolic pathways	54	6.3	2.7E-2	Gbe1, Gch1, Nadk2, Ndufa4l2, Ak4, Aldh1a3, Aldh3a1, Aox1, Aox2, Aldoc, Acer2, Aadat, Alox15, Bco1, Cbr1, LOC102556347, Cers6, Cmpk2, Cyp1a1, Cyp27a1, Cox4i2, Dhrs3, Dgki, Dhodh, Eno2, LOC100911625, Gpi, Gfpt2, Gfpt3, Hpse, Hk2, H6pd, Ipmk, Kyat1, Lpin1, Mboat2, Maob, Mtmr14, Mtmr7, Nampt, Nos2, Olah, Pik3c2b, Pik3c2g, Pfkl, Pla2g2a, Pla2g4f, Pla2g12a, Pygl, P4ha2, Ptges, Pdxp, Spr, LOC497963
HIF-1 signaling pathway	35	4.1	7.2E-7	Pfkfb3, Angpt2, Cdkn1b, Egln1, Egln3, Eno2, LOC100911625, Hmox1, Hk2, Insr, Nos2, Pdk1, LOC497963, Slc2a1, Vegfa, Bnip3, Ndufa4l2, Cmpk2, Ndrg1, Ak4, Ddit4, Ero1a, Ccng2, Maob, Angptl4, Cox4i2, Cbfa2t3, Olah, Higd1a, Fam162a, Foxo3, pdk1, Nampt, HP
PI3K-Akt signaling pathway	22	2.9	4.1E-2	Bcl2l11, Ddit4, Gng4, Mdm2, Angpt2, Col6a5, Col6a6, Col11a2, Csf3, Cdkn1b, Efna1, Fgf11, Fgf9, Foxo3, Gys1, Insr, Il2ra, Il6, Il7, Pdgfra, Reln, Vegfa
FoxO signaling pathway	11	1.3	1.2E-2	Bcl6, Bcl2l11, Bnip3, Fbxo32, Gabarapl1, Mdm2, Ccng2, Cdkn1b, Foxo3, Insr, Il6
TNF signaling pathway	14	1.6	4.1E-5	Ccl5, Cxcl10, Cxcl2, Cx3cl1, Fas, Nfkbia, Tnfaip3, Tnfrsf1b, Traf1, Birc3, Cxcl3, IL1b, Il6, Lif
Glycolysis / Gluconeogenesis	8	0.9	6.8E-3	Aldh1a3, Aldh3a1, Aldoc, Eno2, LOC100911625, Gpi, Hk2, Pfkl

C. shLRP5

Term	Number of genes	%	p-value	Genes
Metabolic pathways	77	6.9	3.2E-4	Gbe1, Papss3, Hmgcs2, Bdh1, Nt5m, Atp6v0a4, Nadk2, Ndufa4l2, Nme3, Ugp2, Acss3, Ak4, Aldh3a1, Aox1, Aox2, Aldoc, Acer2, Aadat, Alox15, Arg1, Bco1, Cbr1, LOC102556347, Cers6, Cdo1, Cyp1a1, Cyp2r1, Cox4i2, Dhrs3, Dck, Dhodh, Eno2, LOC100911625, Gpi, Gls2, Gfpt2, Gatb, Gpat3, Hpse, Hk2, H6pd, Hykk, Hsd11b1, Itpk1, Kyat1, Lpin1, Mboat2, Maob, Mtmr14, Nampt, Nos1, Nos2, Olah, Ogdhl, Pcyt1b, Piga, Pik3c2b, Phospho1, Pfkl, Pla2g2a, Pla2g4b, Pla2g6, Pla2g4f, Pla2g12a, Ppcdc, Pygl, P4ha2, Ptgds, Ptges, Pdxp, Pkm, Spr, LOC497963, Tpi1, LOC500959, LOC100911515, Xylb
HIF-1 signaling pathway	36	3.3	7.2E-7	Pfkfb3, Mknk2, Angpt2, Cdkn1b, Egln1, Egln3, Eno2, LOC100911625, Hmox1, Hk2, Insr, Nos2, Pik3r1, Pdk1, LOC497963, Slc2a1, Vegfa, Bnip3, Ndufa4l2, Cmpk2, Ndrg1, Ak4, Ddit4, Ero1a, Ccng2, Maob, Angptl4, Cox4i2, Cbfa2t3, Olah, Higd1a, Fam162a, Foxo3, pdk1, Nampt, HP
PI3K-Akt signaling pathway	24	2.1	1.3E-2	Bcl2l11, Ddit4, Gng4, Mdm2, Angpt2, Col6a5, Col6a6, Col11a2, Col24a1, Csf3, Cdkn1b, Efna1, Fgf11, Fgf9, Foxo3, Gys1, Insr, Itgb8, Il2ra, Lama5, Pik3r1, Pdgfra, Reln, Vegfa
FoxO signaling pathway	15	1.3	1.5E-3	Bcl6, Bcl2l11, Bnip3, Fbxo32, Gabarapl1, Mdm2, Ccng2, Cdkn1b, Foxo1, Foxo3, Irs3, Insr, Pik3r1, Prkab2, Slc2a4
TNF signaling pathway	10	0.9	3.6E-2	Ccl5, Cxcl2, Cx3cl1, Fas, Tab1, Tnfaip3, Traf1, Birc3, Lif, Pik3r1
Glycolysis / Gluconeogenesis	11	1.0	5.6E-4	Aldh1a3, Aldoc, Eno2, LOC100911625, Gpi, Hk2, Pfkl, Pkm, Tpi1, LOC500959, LOC100911515
